# The Incidence, Risk Factors, and Hospital Mortality of Prolonged Mechanical Ventilation among Cardiac Surgery Patients: A Systematic Review and Meta-Analysis

**DOI:** 10.31083/j.rcm2511409

**Published:** 2024-11-20

**Authors:** Qiaoying Wang, Yuanyuan Tao, Xu Zhang, Shurong Xu, Yanchun Peng, Lingyu Lin, Liangwan Chen, Yanjuan Lin

**Affiliations:** ^1^The School of Nursing, Fujian Medical University, 350005 Fuzhou, Fujian, China; ^2^Department of Nursing, Jingmen Peoples Hospital, 448001 Jingmen, Hubei, China; ^3^Department of Nursing, Women and Children’s Hospital, School of Medicine, Xiamen University, 361005 Xiamen, Fujian, China; ^4^Department of Cardiovascular Surgery, Fujian Medical University Union Hospital, 350001 Fuzhou, Fujian, China; ^5^Department of Nursing, Fujian Medical University Union Hospital, 350001 Fuzhou, Fujian, China

**Keywords:** cardiac surgical patients, in-hospital mortality, meta-analysis, prolonged mechanical ventilation, risk factors

## Abstract

**Background:**

Prolonged mechanical ventilation (PMV) is a common complication after cardiac surgery and is considered a risk factor for poor outcomes. However, the incidence and in-hospital mortality of PMV among cardiac surgery patients reported in studies vary widely, and risk factors are controversial.

**Methods:**

We searched four databases (Web of Science, Cochrane Library, PubMed, and EMBASE) for English-language articles from inception to October 2023. The odds ratio (OR), 95% confidence interval (CI), PMV incidence, and in-hospital mortality were extracted. Statistical data analysis was performed using Stata software. We calculated the fixed or random effects model according to the heterogeneity. The quality of each study was appraised by two independent reviewers using the Newcastle–Ottawa scale.

**Results:**

Thirty-two studies were included. The incidence of PMV was 20%. Twenty-one risk factors were pooled, fifteen risk factors were found to be statistically significant (advanced age, being female, ejection fraction <50, body mass index (BMI), BMI >28 kg/m^2^, New York Heart Association Class ≥Ⅲ, chronic obstructive pulmonary disease, chronic renal failure, heart failure, arrhythmia, previous cardiac surgery, higher white blood cell count, creatinine, longer cardiopulmonary bypass (CPB) time, and CPB >120 min). In addition, PMV was associated with increased in-hospital mortality (OR, 14.13, 95% CI, 12.16–16.41, I^2^ = 90.3%, *p* < 0.01).

**Conclusions:**

The PMV incidence was 20%, and it was associated with increased in-hospital mortality. Fifteen risk factors were identified. More studies are needed to prevent PMV more effectively according to these risk factors.

**The PROSPERO Registration:**

This systematic review and meta-analysis was recorded at PROSPERO (CRD42021273953, https://www.crd.york.ac.uk/prospero/display_record.php?RecordID=273953).

## 1. Introduction

Based on the findings of the Global Burden of Disease study, there was a significant two-fold increase in cardiovascular disease (CVD) prevalence between 1990 and 2019, with the number of afflicted individuals escalating from 271 million to 523 million [1]. The progressive evolution of cardiac surgical technology has engendered a burgeoning cohort of patients eligible for surgical interventions. Despite the advancements in perioperative management, anesthesia, cardiopulmonary bypass (CPB), and the surgical environment remain vulnerable to postoperative functional changes, which give rise to consequential complications. Among these, the incidence of prolonged mechanical ventilation (PMV) constitutes a substantial proportion, accounting for up to 53.27% [2]. PMV has been correlated with heightened reintubation and prolonged duration of stay in the intensive care unit (ICU), leading to more pulmonary complications [3].

Globally, the number of PMVs has increased. Within the United States, the demand for PMVs reached an estimated 625,000 cases in 2020, while in Taiwan, 92,324 patients required PMVs between 2015 and 2019 [4, 5, 6]. PMV not only results in negative patient experiences but also reduces their quality of life. The complications arising from PMV, encompassing muscle wasting, functional impairment, and diaphragmatic dysfunction, have been found to be associated with extended hospitalization periods and elevated mortality. Consequently, these complications impose considerable financial strain upon afflicted families [7, 8, 9, 10, 11]. The reported incidence of PMV in patients undergoing cardiac surgery ranges from 1.96% to 53.27% [2, 10, 12, 13, 14], and the risk factors for PMV include older age [12, 15], emergency surgery [10, 15, 16], and being female [9, 15]. However, these findings are controversial. The current evidence on PMV prevention and treatment in cardiac surgical patients has yet to be comprehensively synthesized, largely due to variations in PMV definitions and study designs across different investigations. In addition, the effect of PMV on in-hospital mortality remains controversial [8, 14]. Therefore, identifying PMV-related risk factors and prognoses may optimize clinical treatment and decision-making to assist surgeons in surgical planning and postoperative management.

Thus, through a systematic review and meta-analysis, the present synthesis endeavors to investigate the incidence, identify risk factors and analyze in-hospital mortality in patients experiencing PMV after cardiac surgical procedures. The overarching objective of this study is to furnish evidence that informs the prevention and effective management of PMV-associated complications in this specific patient population.

## 2. Methods

This systematic review and meta-analysis were conducted per the Preferred Reporting Items for Systematic Reviews and Meta-Analysis (PRISMA) guidelines (**Supplementary material 1**) and recorded at PROSPERO.

### 2.1 Search Methods

Databases, including PubMed, Web of Science, EMBASE, and the Cochrane Library, were independently searched by two researchers for studies published from the database inception through October 2023. The retrieval strategy combined cardiac surgery with PMV. Detailed search strategies are shown in **Supplementary material 2**.

Two researchers independently selected the articles. First, duplicate studies were removed by EndNote X20 (Thomson ResearchSoft, Philadelphia, PA, USA), and inappropriate studies were eliminated based on the title and abstract. After screening by title and abstract, we assessed the articles to select qualified literature through full-text screening. Disputes or disagreements were resolved through discussions with a third researcher.

The inclusion criteria were as follows: age: 18 years or older; undergoing cardiac surgery; data should be available for extracting the incidence, risk factors, or in-hospital mortality for PMV; risk factors for PMV must be assessed by an odds ratio (OR) with a 95% confidence interval (CI); PMV was defined as a ventilation time ≥24 hours (h); study types included case–control trials and cohort studies. The exclusion criteria were as follows: case reports, protocols, commentaries, letters or abstracts, and high-risk bias literature (Newcastle–Ottawa Quality Assessment scale ≤4).

### 2.2 Quality Appraisal

The quality of the included studies was independently appraised and cross-checked by two reviewers using the Newcastle–Ottawa Quality Assessment scale (NOS). The total score for NOS is 9. The risk of bias was classified into three categories: low-quality, NOS ≤4, which should be excluded from the meta-analysis; moderate-quality, NOS 5–6; high-quality, NOS ≥7. Any inconsistent evaluation was discussed with the third researcher.

According to the Grading of Recommendations Assessment, Development, and Evaluation (GRADE) methodology, two investigators independently assessed the quality of the evidence for risk factors. Evidence grades were divided into the following categories: high, where authors were confident that the estimated effect was similar to the actual effect; moderate, where the estimated effect was probably close to the exact effect; low, where the true effect might be different from the estimated effect; very low, where the actual effect was probably markedly different from the estimated effect.

### 2.3 Data Abstraction

Two researchers independently extracted data from the included studies using a pre-designed data form. The following data were extracted: the characteristics of each study, risk factors, and in-hospital mortality.

### 2.4 Synthesis

We used Stata version 17.0 (StataCorp LLC, College Station, TX, USA) to perform a meta-analysis. The incidences of PMV, in-hospital mortality, and PMV-related risk factors were pooled. Subgroup analyses were performed according to PMV definitions and study population. OR and 95% CI were calculated to assess the strength of the associations, and Cochran’s Q and I^2^ tests were used to evaluate heterogeneity. I^2^< 50% indicated no statistical heterogeneity between studies, and a fixed effect model (FD) was used for data analysis. I^2^ > 50% indicated statistical heterogeneity between studies; a random effect model (RD) was used, and a subgroup analysis was performed to explore the source of heterogeneity. I^2^ between 0% and 25% represented low heterogeneity, I^2^ between 25% and 50% was moderate heterogeneity, and I^2^ > 50% indicated high heterogeneity. Point estimate differences, where the 95% CI did not overlap with 1, were considered statistically significant at *p* < 0.05. We performed a sensitivity analysis when the meta-analysis involved more than four studies. Funnel plots were also performed when more than ten studies were involved.

## 3. Results

### 3.1 General Characteristics of the Studies

**Fig. 1.**
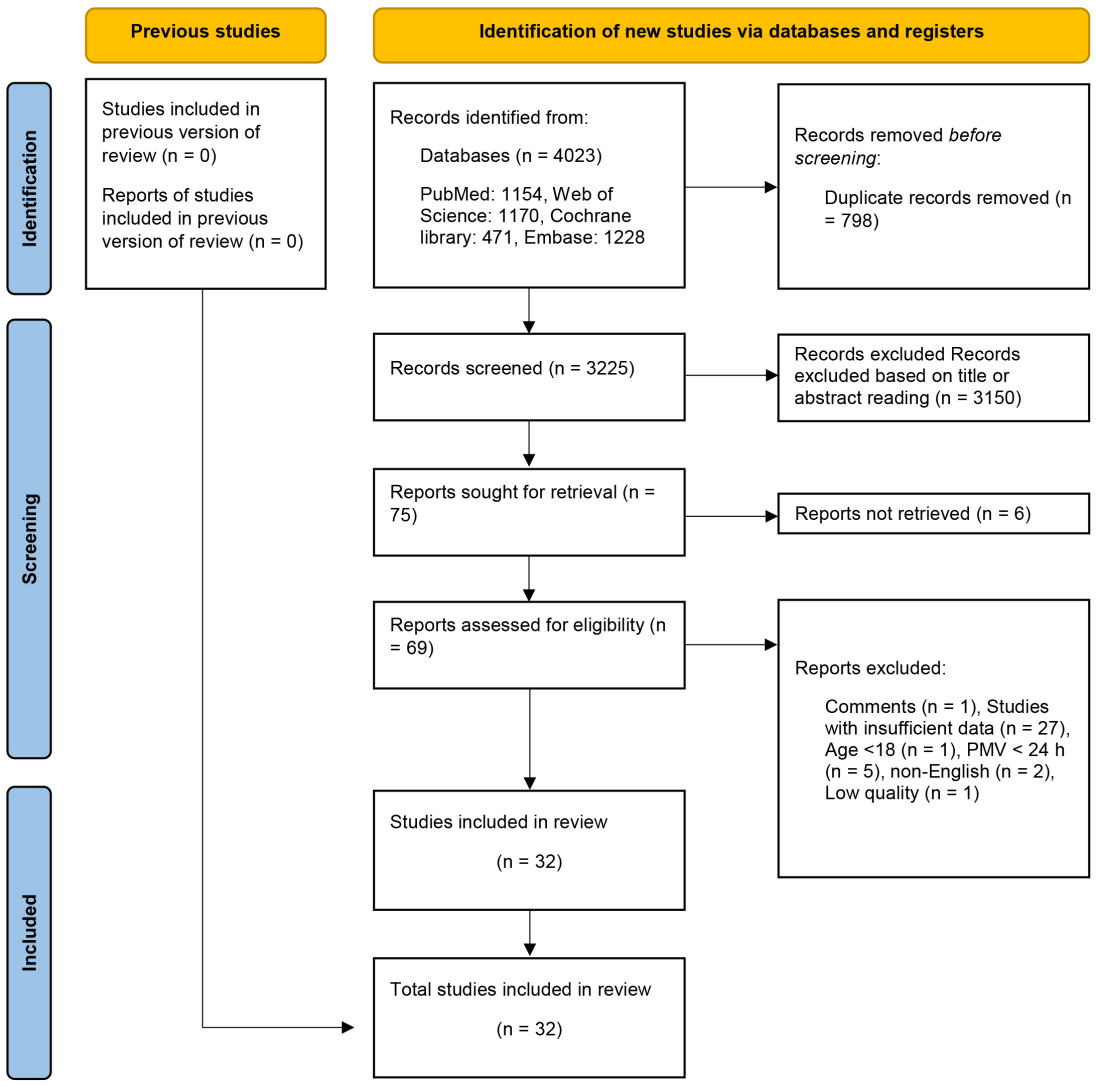
**Flow diagram of research strategy**. PMV, prolonged mechanical ventilation.

After eliminating duplicate entries, 3325 articles underwent preliminary screening through title and abstract assessments. Following this, seventy-two eligible articles were subjected to full-text screening. After conducting a thorough full-text screening, forty-two articles were excluded from the analysis. Among these, twenty-seven studies were excluded due to insufficient data: one was a review, one had participants below the age of 18 years, five had a PMV <24 h, six were not available in full text, and two were non-English literature. Additionally, one article of low quality was excluded. Finally, thirty-two studies were deemed suitable for inclusion in the quantitative analysis (Fig. 1). The categorization of PMV varied among the included studies. Specifically, twelve studies defined PMV as ≥24 h, ten studies defined it as ≥48 h, six studies described it as ≥72 h, and one article investigated PMV at 24 h, 48 h, and 72 h. The general characteristics of the studies are summarized in Table 1 (Ref. [2, 7, 8, 9, 10, 12, 13, 14, 15, 16, 17, 18, 19, 20, 21, 22, 23, 24, 25, 26, 27, 28, 29, 30, 31, 32, 33, 34, 35, 36, 37, 38]).

**Table 1.** **Characteristics of the included studies**.

Author/year	Country	Study design	Age		Study population	Sample Size	PMV definition	PMV incidence	Mortality	
			PMV	Non-PMV					PMV	Non-PMV
Engle J *et al*. 1999 [17]	America	Retrospective case–control study	NA	NA	TAAA	256	≥72 h	41.8%	NA	NA
Kern H *et al*. 2001 [18]	Germany	Retrospective cohort study	Median: 66.2	Median: 62.3	CABG, valve	687	≥48 h	9%	11.3%	0.48%
Légaré JF *et al*. 2001 [19]	Canada	Retrospective cohort study	65.40 ± 10.60		CABG	1829	≥24 h	8.6%	18.5%	1.2%
Yende S *et al*. 2004 [20]	America	Retrospective cohort study	NA	NA	CABG	400	≥24 h	6.75%	NA	NA
Natarajan K *et al*. 2006 [21]	India	Retrospective cohort study	56.80 ± 9.40	56.90 ± 8.80	CABG	470	≥24 h	4.7%	36.3%	0.45%
Lei Q *et al*. 2009 [22]	China	Retrospective cohort study	48.90 ± 10.70	44.20 ± 10.70	Aortic arch surgery	255	≥72 h	10.2%	11.5%	0.9%
Shirzad M *et al*. 2010 [23]	Iran	Retrospective cohort study	53.93 ± 12.84	48.25 ± 13.66	Valve surgery	1056	≥24 h	6.6%	42.9%	2.2%
Christian K *et al*. 2011 [24]	America	Retrospective cohort study	66.00 ± 11.10	66.40 ± 10.80	CABG	464	≥72 h	25%	12.9%	2.9%
Piotto RF *et al*. 2012 [16]	Brazil	Retrospective cohort study	62.00 ± 9.50	67.30 ± 9.10	CABG	3010	≥48 h	2.6%	58.4%	2.3%
Siddiqui MMA *et al*. 2012 [25]	Pakistan	Retrospective cohort study	39.50 ± 21.28	30.29 ± 13.95	CABG, valve	1617	≥24 h	4.8%	32.5%	0.4%
Saleh HZ *et al*. 2012 [12]	UK	Retrospective cohort study	68.6 (63.2, 73.7)	65.2 (58.5, 71.1)	CABG	10,977	≥72 h	2.0%	28.4%	0.5%
Bartz RR *et al*. 2015 [13]	UK	Retrospective cohort study	65.20 ± 11.90	62.60 ± 12.70	CABG	3881	≥48 h	33.2%	NA	NA
Gumus F *et al*. 2015 [8]	Turkey	Retrospective cohort study	65.60 ± 9.30	60.40 ± 9.90	CABG	830	≥24 h	5.6%	45.7%	4%
Sharma V *et al*. 2017 [15]	Canada	Retrospective cohort study	65 (37, 80)	67 (47, 81)	Valve surgery	21,654	≥48 h	6.2%	NA	NA
Wise ES *et al*. 2017 [26]	America	Retrospective cohort study	64.35 ± 3.35	62.95 ± 3.90	CABG	738	≥24 h	14.1%	NA	NA
Chen Y *et al*. 2019 [9]	China	Retrospective cohort study	58.30 ± 10.92	50.94 ± 10.84	AAAD	102	≥72 h	29.4%	20.7%	5.6%
Hsu H *et al*. 2019 [10]	Taiwan, China	Retrospective cohort study	64.00 ± 11.00		CABG	382	≥48 h	11.3%	NA	NA
Papathanasiou M *et al*. 2019 [7]	Germany	Retrospective cohort study	58.5 ± 10.5	57.5 ± 10.5	LVAD	139	≥7 d	43.2%	60.0%	2.5%
Aksoy R *et al*. 2021 [27]	Turkey	Retrospective cohort study	60.80 ± 10.20	59.20 ± 10.60	CPB	207	≥24 h	20.8%	37.2%	5.5%
Ge M *et al*. 2021 [14]	China	Retrospective cohort study	53.60 ± 12.40	51.10 ± 12.80	De Bakey type I aortic dissection	582	≥48 h	44.5%	18.1%	7.4%
Kreibich M *et al*. 2022 [28]	Germany	Retrospective cohort study	68.4 (9.9)		Valve surgery	2597	≥48 h	16.3%	NA	NA
Lin L *et al*. 2022 [2]	China	Retrospective cohort study	52.5 ± 11.7		AAAD	734	≥48 h	53.3%	NA	NA
Li X *et al*. 2022 [29]	China	Retrospective cohort study	58 (50, 65)	54 (46, 62)	Cardiac surgery	3919	≥48 h	13.6%	3.8%	0.5%
Michaud L *et al*. 2022 [30]	France	Retrospective cohort study	67.3 ± 12	67.4 ± 11.5	CPB	568	≥24 h	12.0%	47.1%	4.6%
Meng Y *et al*. 2023 [31]	China	Retrospective cohort study	61.44 ± 12.04	59.33 ± 12.08	Cardiac surgery	693	≥24 h	21.2%	NA	NA
Sankar A *et al*. 2022 [32]	Canada	Retrospective cohort study	71 (11)	73 (10)	CPB	4809	≥24 h	15.0%	14.0%	1.0%
Xie Q *et al*. 2022 [33]	China	Retrospective cohort study	49.86 ± 11.123;	44.87 ± 9.444;	AAAD	384	≥24 h	55.5%	NA	NA
			51.40 ± 10.908;	45.58 ± 10.003;			≥48 h	35.4%		
			51.72 ± 10.446	46.28 ± 10.435			≥72 h	25.0%		
Xiao Y *et al*. 2022 [34]	China	Retrospective cohort study	54.5 (39.0, 63.0)	64 (56.0, 69.5)	Redo valve surgery	117	≥24 h	38.5%	13.3%	0%
Zhang Q *et al*. 2023 [35]	China	Retrospective cohort study	NA	NA	Cardiac surgery	3835	≥48 h	NA	NA	NA
Shen X *et al*. 2022 [36]	China	Retrospective cohort study	66 (55, 72)	66 (54, 71)	CPB	118	≥48 h	45.8%	NA	NA
Rahimi S *et al*. 2023 [37]	Iran	Retrospective cohort study	NA	NA	CABG	1361	≥24 h	21.4%	NA	NA
Zhou Y *et al*. 2023 [38]	China	Retrospective cohort study	65 (57, 75)	52 (47, 62)	Valve surgery	105	≥72 h	40%	7.1%	0%

NA, not applicable; TAAA, Stanford type A aortic dissection; CABG, coronary artery bypass graft; UK, United Kingdom; AAAD, acute type A aortic dissection; LVAD, left ventricular assist device; CPB, cardiopulmonary bypass; PMV, prolonged mechanical ventilation; h, hours; d, days.

### 3.2 Risk of Bias

Based on NOS, three studies scored 8, fifteen scored 7, and eleven scored 6. These assessments revealed that 62% of the included studies satisfied the standards for high quality (**Supplementary material 3**).

### 3.3 Syntheses of Results

#### 3.3.1 Incidence of PMV

**Fig. 2.**
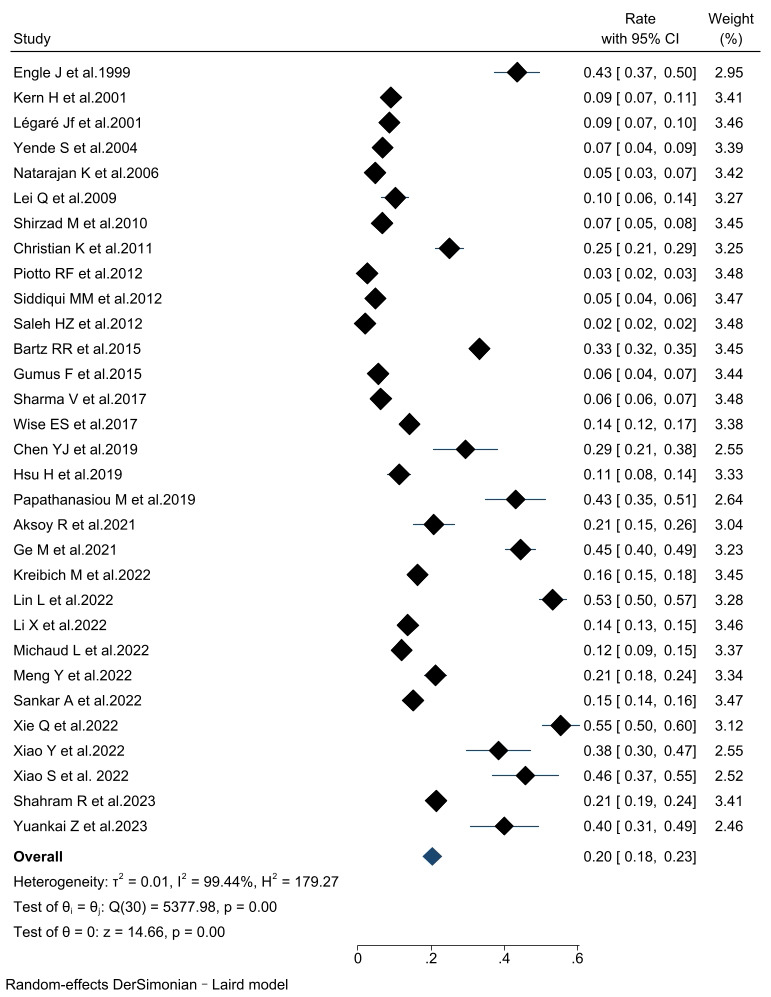
**The forest plot of prolonged mechanical ventilation incidence**. CI, confidence interval.

**Fig. 3.**
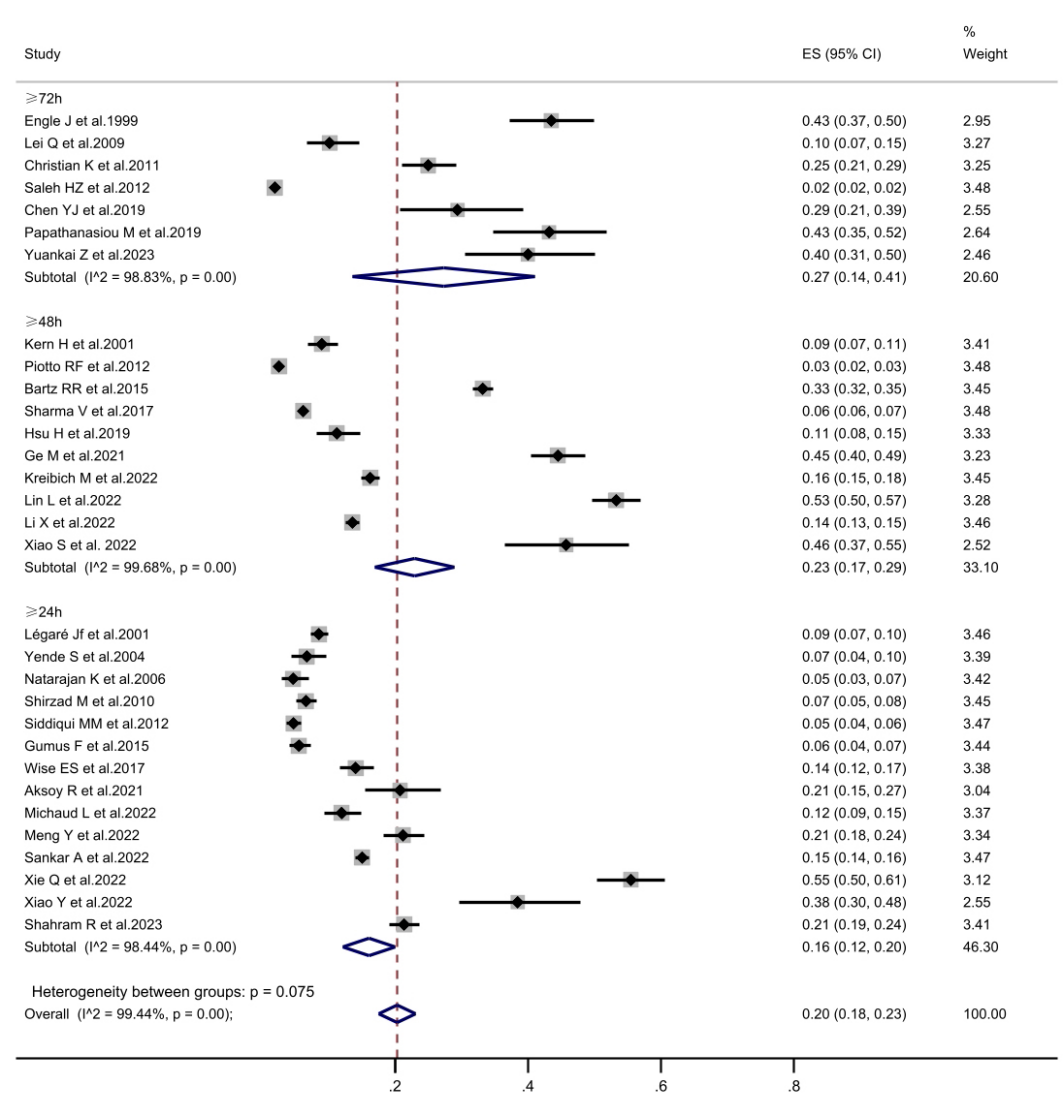
**The forest plot of subgroup analysis in prolonged mechanical ventilation incidence is defined according to prolonged mechanical ventilation**. CI, confidence interval; ES, Eric Stephen Wise.

**Fig. 4.**
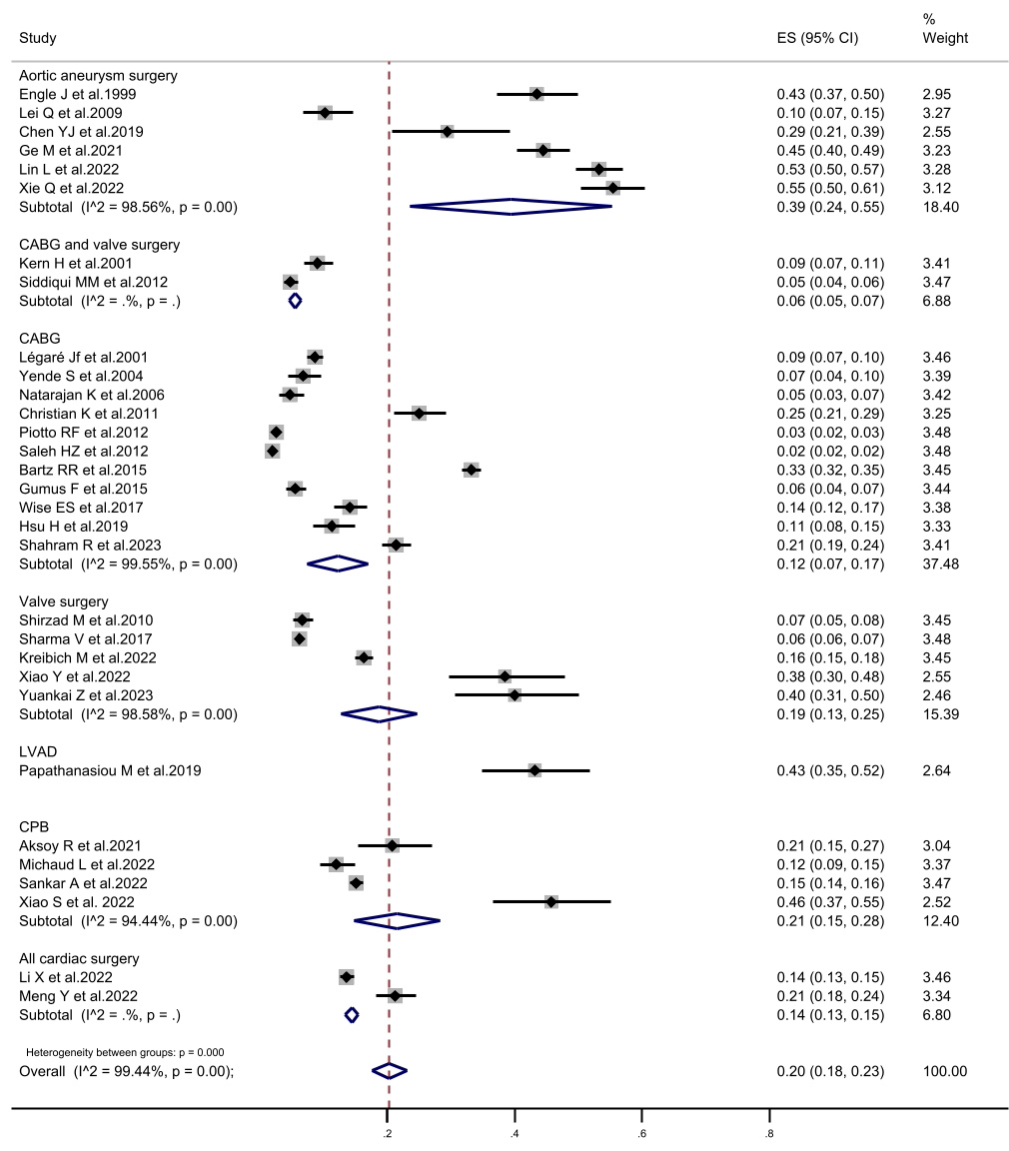
**The forest plot of subgroup analysis in prolonged mechanical ventilation incidence according to the study population**. CI, confidence interval; CABG, coronary artery bypass grafting; LVAD, left ventricular assist device; CPB, cardiopulmonary bypass; ES, Eric Stephen Wise.

The incidence of PMV in patients undergoing cardiac surgery was 20% (95% CI, 18%–23%) across the thirty-two included articles. Subgroup analyses were performed according to the PMV definition and the study population (Fig. 2). Consistent findings were observed throughout all subgroup analyses. The combined incidence of PMV was 16.1% (95% CI, 12.1%–20.1%) in the PMV ≥24 h group, 23.0% (95% CI, 17.0%–28.9%) in the PMV ≥48 h group; 27.3% (95% CI, 13.6%–41.0%) in the PMV ≥72 h group (Fig. 3). Six studies examining aortic surgery were combined, revealing a PMV incidence of 39.4% (95% CI, 23.5%–55.3%). Two studies combining coronary artery bypass grafting (CABG) with valve surgery had a PMV incidence of 5.6% (95% CI, 4.6%–6.5%), while eleven studies examining CABG alone had an incidence of 12.2% (95% CI, 7.5%–17.0%). Five studies investigating valve surgery alone had an incidence of 18.7% (95% CI, 12.7%–24.7%), and four studies examining cardiac surgery under CPB had an incidence of 21.5% (95% CI, 14.7%–28.2%). Finally, two studies enrolling all types of patients undergoing cardiac surgery had an incidence of 14.4% (95% CI, 13.4%–15.5%) (Fig. 4).

#### 3.3.2 The Risk Factors for PMV

Eighteen preoperative risk factors were synthesized, with advanced age (OR, 1.03, 95% CI, 1.02–1.04, I^2^ = 53.9%, *p* < 0.01), being female (OR, 1.68, 95% CI, 1.18–2.39, I^2^ = 83.3%, *p* < 0.01), ejection fraction (EF) <50 (OR, 2.35, 95% CI, 1.80–3.09, I^2^ = 60.8%, *p* < 0.01), body mass index (BMI) (OR, 1.07, 95% CI, 1.00–1.14, I^2^ = 79.9%, *p* = 0.03), BMI >28 kg/m^2^ (OR, 2.24, 95% CI, 1.74–2.87, I^2^ = 14.5%, *p* < 0.01), New York Heart Association Class (NYHA) ≥Ⅲ (OR, 2.01, 95% CI, 1.41–2.87, I^2^ = 77.8%, *p* < 0.01), chronic obstructive pulmonary disease (COPD) (OR, 1.61, 95% CI, 1.37–1.90, I^2^ = 16.3%, *p* < 0.01), chronic renal failure (OR, 2.55, 95% CI, 1.98–3.29, I^2^ = 39.4%, *p* < 0.01), heart failure (OR, 3.62, 95% CI, 1.31–10.05, I^2^ = 87.6%, *p* = 0.01), arrhythmia (OR, 1.87, 95% CI, 1.07–3.29, I^2^ = 87.2%, *p* < 0.01), previous cardiac surgery (OR, 1.96, 95% CI, 1.65–2.34, I^2^ = 30.6%, *p* < 0.01), higher white blood cell count (WBC) (OR, 1.11, 95% CI, 1.04–1.18, I^2^ = 23.7%, *p* < 0.01), and creatinine (OR, 1.01, 95% CI, 1.00–1.02, I^2^ = 27.1%, *p* < 0.01) being identified as statistically significant (**Supplementary material 4**). Conversely, hypertension (OR, 1.17, 95% CI, 0.91–1.50, I^2^ = 75.5%, *p* = 0.23), diabetes (OR, 1.31, 95% CI, 1.00–1.72, I^2^ = 76.9%, *p* = 0.09), vascular lesions ≥3 (OR, 2.04, 95% CI, 0.98–4.22, I^2^ = 96.2%, *p* = 0.06), emergency surgery (OR, 1.77, 95% CI, 0.41–7.68, I^2^ = 94.4%, *p* = 0.44), and suffering a previous stroke (OR, 1.44, 95% CI, 0.88–2.35, I^2^ = 78.2%, *p* = 0.15) did not demonstrate statistically significant associations with PMV incidence (Table 2A,2B,2C and **Supplementary material 4**).

**Table 2A.** **Forest plot results - Risk factors**.

Name	Number of articles included	OR	95% CI	I^2^ (%)	*p*
Advanced aged	12	1.03	1.02–1.04	53.9	<0.01
Being female	7	1.68	1.18–2.39	83.3	<0.01
Ejection fraction <50	6	2.32	1.72–3.13	66.6	<0.01
Body mass index	4	1.07	1.01–1.14	79.9	0.03
Body mass index >28	3	2.24	1.74–2.87	14.5	<0.01
NYHA ≥III	5	2.01	1.41–2.87	77.8	<0.01
COPD	6	1.61	1.37–1.90	16.3	<0.01
Chronic renal failure	8	2.47	1.92–3.19	13.0	<0.01
Heart failure	2	3.14	0.79–12.55	89.3	0.01
Arrhythmia	3	1.87	1.07–3.29	87.2	<0.01
Previous cardiac surgery	8	1.98	1.75–2.23	30.6	<0.01
White blood cell count	4	1.11	1.06–1.17	0	<0.01
Creatinine	4	1.01	1.00–1.02	27.1	<0.01
Hypertension	5	1.11	0.90–1.36	68.4	0.23
Diabetes	7	1.31	1.00–1.72	76.9	0.09
Three or more vessel disease	3	2.04	0.98–4.22	96.2	0.06
Emergency surgery	6	1.78	0.41–7.68	94.4	0.44
Perioperative stroke	3	1.15	0.81–1.62	67.2	0.15
Longer CPB time	8	1.03	1.01–1.05	86.3	0.02
CPB time >120 min	2	3.16	1.25–7.95	61.9	<0.01
Cross-clamp time	5	1.00	0.99–1.02	81.1	0.50

**Table 2B.** **Forest plot results - Subgroup analysis**.

**Study population**					
Name	Study population	Number of articles included	OR	95% CI	I^2^ (%)
Advanced aged	Aortic aneurysm surgery	4	1.03	1.02–1.04	0
	CABG	4	1.04	1.02–1.06	68.7
	Valve surgery	3	1.57	0.99–1.11	70.4
	All cardiac surgeries	1	1.03	1.02–1.04	-
Ejection fraction <50	CABG	3	2.93	1.16–7.39	80.9
	Others	3	2.23	1.70–2.92	54.6
Body mass index	CABG	2	1.04	0.93–1.16	90.2
	Aortic aneurysm surgery	2	1.10	1.06–1.15	0
Hypertension	Aortic aneurysm surgery	1	0.81	0.38–1.07	-
	CABG	1	1.48	1.08–2.02	-
	Valve surgery	2	0.97	0.64–1.47	87.4
	CPB	1	1.22	0.99–1.50	-
Diabetes	CABG	3	1.68	1.12–2.53	78.1
	Non-CABG	3	1.01	0.72–1.41	64.4
COPD	CABG	4	1.83	1.36–2.47	0
	Aortic aneurysm surgery	2	1.52	1.25–1.85	53.0
Chronic renal failure	CABG	6	2.68	1.88–3.82	23.4
	CABG and valve surgery	1	2.43	1.32–4.49	-
	LVAD	1	1.15	0.34–4.05	-
Longer CPB	Aortic aneurysm surgery	3	1.01	1.00–1.02	0
	Others	2	1.02	0.99–1.05	46.2
**PMV definition**					
Name		Number of articles included	OR	95% CI	I^2^ (%)
Advanced aged	PMV ≥24	3	1.03	0.98–1.08	76.4
	PMV ≥48	6	1.03	1.02–1.04	59.6
	PMV ≥72	3	1.04	1.02–1.05	0
Ejection fraction <50	PMV ≥24	3	2.83	1.49–5.36	79.5
	PMV ≥48	3	2.20	1.51–3.19	60.0
Being female	PMV ≥24	2	1.80	1.30–2.51	0
	PMV ≥48	3	1.10	0.87–1.47	73.8
	PMV ≥72	2	4.14	2.39–7.20	0
Hypertension	PMV ≥24	1	1.48	1.08–2.02	-
	PMV ≥48	3	0.94	0.66–1.34	76.6
	PMV ≥72	1	1.48	1.08–2.02	-
Diabetes	PMV ≥24	2	1.41	0.83–2.42	58.7
	PMV ≥48	3	1.41	0.94–2.10	86.6
	PMV ≥72	2	0.83	0.19–3.61	85.7
COPD	PMV ≥24	2	1.70	1.17–2.48	15.8
	PMV ≥48	4	1.59	1.32–1.91	36.0
Chronic renal failure	PMV ≥24	5	2.22	1.65–2.99	0
	PMV ≥48	3	3.13	1.49–6.58	47.5
Emergency surgery	PMV ≥24	3	2.05	0.15–27.37	88.0
	PMV ≥48	3	1.51	0.26–8.71	95.3
Previous cardiac surgery	PMV ≥24	2	2.17	1.12–4.17	35.9
	PMV ≥48	5	1.91	1.60–2.28	30.1
	PMV ≥72	1	5.08	1.67–15.43	-
Longer CPB	PMV ≥24	3	2.58	0.78–8.50	89.1
	PMV ≥48	4	1.01	1.00–1.02	0
	PMV ≥72	2	1.15	0.96–1.37	92.9

**Table 2C.** **Forest plot results - In-hospital mortality**.

Name			Number of articles included	OR	95% CI	I^2^ (%)
Overall mortality			17	14.13	12.16–16.41	90.3
Subgroup analysis	Study population	CABG	4	26.51	20.58–34.14	94.2
		Valve surgery	3	18.90	11.69–30.55	0
		CABG and valve surgery	2	34.67	16.84–71.40	92.6
		Aortic aneurysm surgery	3	2.95	1.95–4.47	52.4
		Cardiopulmonary bypass surgery	3	11.94	9.11–15.65	51.4
		Others	2	8.69	4.90–15.42	59.0
	PMV definition	PMV ≥24	8	14.88	12.15–18.23	76.1
		PMV ≥48	3	3.60	2.51–5.15	72.0
		PMV ≥72	6	30.02	22.59–39.90	89.2

OR, odds ratio; CI, confidence interval; NYHA, New York Heart Association (classification); COPD, chronic obstructive pulmonary disease; CPB, cardiopulmonary bypass; CABG, coronary artery bypass grafting; PMV, prolonged mechanical ventilation; LVAD, left ventricular assist device.

Three intraoperative risk factors were identified and summarized. Our analysis revealed that longer CPB time and CPB time >120 min were associated with increased risk factors of PMV (CPB: OR, 1.03, 95% CI, 1.01–1.05, I^2^ = 86.3%, *p* = 0.02; CPB >120 min: OR, 2.52, 95% CI, 1.82–3.48, I^2^ = 24.4%, *p* < 0.01), while aortic cross-clamp time (OR, 1.00, 95% CI, 0.99–1.02, I^2^ = 81.1%, *p *= 0.50) did not demonstrate a statistically significant association. In addition, significant heterogeneity was observed in 14 of the 21 examined risk factors; four exhibit low heterogeneity, while the remaining three demonstrate moderate heterogeneity (Table 2A,2B,2C and **Supplementary material 4**).

Subgroup analyses were performed for each risk factor according to the PMV definition and study population. The pooled effect and heterogeneity remained largely unchanged across various subgroups, including advanced age, being female, EF <50, higher BMI, hypertension, diabetes, COPD, chronic renal failure, emergency surgery, previous cardiac surgery, previous stroke, longer CPB time, and aortic cross-clamp time. Diagrams of the risk factor analysis in **Supplementary material 4**.

A sensitivity analysis was performed for several risk factors, including advanced age, being female, EF <50, higher BMI, NYHA ≥III, hypertension, diabetes, COPD, chronic renal failure, emergency surgery, previous cardiac surgery, previous stroke, higher WBC, creatinine, longer CPB time, and aortic cross-clamp time. The analysis demonstrated that the pooled effect of hypertension and diabetes were significantly changed (hypertension: OR, 1.28, 95% CI, 1.02–1.61, I^2^ = 60.5%, *p *= 0.03; diabetes: OR, 1.42, 95% CI, 1.11–1.81, I^2^ = 73.2%, *p* < 0.01) (**Supplementary material 3**).

#### 3.3.3 The In-Hospital Mortality of PMV

Seventeen out of the thirty-two studies reported data on in-hospital mortality in both the PMV and non-PMV groups, with our analysis revealing a significant association between PMV and in-hospital mortality (OR, 14.13, 95% CI, 12.16–16.41, I^2^ = 90.3%, *p* < 0.01). Subgroup analyses based on different study populations and PMV definitions yielded consistent results, with PMV being most strongly associated with in-hospital mortality in the PMV ≥72 h group and CABG with valve surgery group (PMV ≥72 h: OR, 30.02, 95% CI, 22.59–39.90, I^2^ = 89.2%, *p* < 0.01; CABG with valve surgery: OR, 34.67, 95% CI, 16.82–71.40, I^2^ = 92.6%, *p* < 0.01) (Table 2A,2B,2C and **Supplementary material 4**).

#### 3.3.4 Quality of the Evidence

The findings indicate that advanced age, being female, COPD, chronic renal failure, and higher WBC exhibited a high level of supporting evidence classified as high-quality; EF <50, BMI >28, NYHA ≥III, and CPB >120 min were supported by moderate-quality evidence. The remaining risk factors were supported by low-quality evidence (**Supplementary material 3**).

## 4. Discussion

This systematic review and meta-analysis represent the inaugural endeavor to comprehensively examine the incidence, risk factors, and in-hospital mortality concerning PMV in cardiac surgery patients. The synthesis included 32 studies involving a total of 68,766 patients and yielded the subsequent key findings: PMV incidence was 20%; 15 risk factors were associated with PMV (advanced age, being female, EF <50, higher BMI, BMI >28, NYHA ≥III, COPD, chronic renal failure, heart failure, arrhythmias, previous cardiac surgery, higher WBC, creatinine, longer CPB time, and CPB time >120 min); PMV was associated with increased in-hospital mortality.

PMV is a well-recognized complication of cardiac surgery, with an incidence ranging from 1.96% to 53.27% [12, 14]. The observed variation in reported incidences of PMV may be attributed to the lack of standardized definitions across studies. To address this issue and enhance the homogeneity of the study population, we employed a uniform definition of PMV as a duration equal to or exceeding 24 hours. By implementing this consistent criterion, we aimed to mitigate potential discrepancies in the identification and classification of PMV cases, ensuring a more reliable and comparable analysis of relevant outcomes. CVD patients are typically extubated within 6 h of surgery; 24 h is sufficient to stabilize their hemodynamics and eliminate the negative effects of surgery and CPB [25, 39]. Concurrently, PMV ≥24 h also aligns with current clinical practice guidelines recommending early extubation [40]. Of paramount significance is the notable reduction in the potential for underestimating the incidence of PMV.

The pooled incidence of PMV among patients who underwent cardiac surgery was determined to be 20%. Subgroup analysis, according to the study population, revealed that the highest incidence of PMV was 39.4% in aortic surgical patients (patients with aortic dissection and aortic arch disease). Conversely, the lowest incidence of PMV was observed in patients undergoing CABG combined with valve surgery, with a rate of 5.6%. In the subgroup analysis of PMV definitions, when PMV was defined as a duration of 72 hours or longer, the incidence was higher at 27.3%, compared to definitions of 24 h (16.1%) and 48 h (23.0%). Notably, the PMV ≥72-hour group primarily consisted of patients undergoing aortic surgery and those with a left ventricular assist device (LVAD), constituting the main study subjects within this particular subgroup. Aortic dissection is a catastrophic cardiovascular clinical event associated with high mortality and surgical risk [41]. Compared to other CVD patients, patients undergoing aortic surgery are more prone to postoperative complications and ischemia-reperfusion injury, which leads to perioperative hemodynamic instability [42, 43, 44]. In the case of patients with LVAD, in addition to the severity of the heart failure syndrome, the considerable burden of comorbidities places them at a high risk of postoperative complications [7]. Furthermore, most studies in the PMV ≥72 h group had small sample sizes, which might have resulted in an imprecise assessment of the incidence of PMV. The PMV ≥24 h group had the lowest incidence of PMV. Despite the fact that the study population of the six included publications originated from developing countries, we posit that defining PMV as a duration of 24 h or more neither overestimates nor underestimates the incidence of PMV. This assertion is based on several factors, including the substantial sample sizes employed in each study, the current contemporary intensive care management practices, and the notable advancements in clinical care and prognoses observed among patients in developing countries.

Evaluating the risk factors associated with PMV in patients undergoing cardiac surgery is essential for improving patient prognosis. In our synthesis, we identified advanced age, being female, and BMI as demographic characteristics that exhibited a significant association with PMV and served as risk factors. Patients who developed PMV in the included articles were predominantly over 60 years of age. Advanced age would reduce functional reserves and increase comorbidities, which have been associated with PMV. The assessment of other surgical patients provided the same conclusion [45, 46]. Although the relationship between PMV and women continues to receive widespread attention, the findings remain controversial. Epidemiological studies have consistently provided evidence supporting the protective role of estrogen against CVD in women. Notably, before menopause, the incidence of coronary heart disease is lower in women compared to men. This observation is attributed, at least in part, to the beneficial effects of estrogen on various cardiovascular parameters, including lipid metabolism, vascular function, and inflammation [47]. Therefore, we speculated that there may have been a selection bias. In addition, being female has been identified as a possible risk factor for intensive care unit-acquired weakness, which causes diaphragmatic weakness and prolongs the duration of mechanical ventilation [48, 49]. Whether a higher BMI is associated with PMV has been debated. Our results suggest a higher BMI and BMI >28 kg/m^2^ are risk factors. However, the evidence for a higher BMI was low; only three publications mentioned a BMI >28 kg/m^2^. Coincidentally, the latest meta-analysis on the impact of BMI on patients undergoing cardiac surgery concluded that being underweight was a predictor of worse survival outcomes, whereas a slightly higher BMI was a protective factor [50]. Therefore, further investigation is required to determine whether BMI causally affects PMV. It is imperative to investigate the incidence of PMV across different weight categories, including underweight, normal weight, overweight, and obese patients undergoing cardiac surgery. In summary, a comprehensive evaluation of patient demographics and characteristics before surgery is important to create an individualized care plan and manage the airway, which reduces PMV.

The influence of heart conditions cannot be ignored. Ejection fraction <50, NYHA ≥III, heart failure, and arrhythmias were also preoperative risk factors for PMV. A lower EF has a negative impact on the occurrence of PMV, which results in higher ventricular filling pressures and lower cardiac output that cause hemodynamic instability and postoperative complications [13, 51]. However, NYHA ≥III, heart failure, and arrhythmias showed low evidence and high heterogeneity. When we performed subgroup analysis based on the PMV definition, the heterogeneity was significantly lower in NYHA ≥III than before. Moreover, the OR was higher in the PMV ≥24 h group than in the PMV ≥48 h group; thus, defining PMV as ≥24 h may help identify risk factors more adequately. Furthermore, preoperative optimization of a patient’s cardiac condition plays an important role in preventing the adverse effects of PMV.

In addition, we confirmed that the patient’s medical history and information on other diseases are crucial for preventing PMV. COPD, chronic renal failure, previous cardiac surgery, higher WBC, and creatinine were proved. Comorbidities such as renal injury and pulmonary hypertension are worthy of attention as they increase the risk of severe cardiac dysfunction and limit physiologic reserve [52, 53]. Cardiac patients, such as those with renal impairment, often have other comorbidities (e.g., diabetes and hypertension) that increase their mental, physiological, and economic burden. The same conclusion has been reported in previous studies [54, 55]. While our analysis suggested that hypertension and diabetes mellitus do not significantly elevate the risk of PMV, it is crucial to acknowledge that the evidence supporting this conclusion was of low quality. Consequently, further research with higher-quality evidence is warranted to establish the relationship between these comorbidities and PMV risk definitively.

Regarding intraoperative risk factors for PMV, longer CPB times and CPB >120 min were identified. The same results were obtained when different study populations and PMV definitions were used for the subgroup analyses. Extended CPB duration precipitates a cascade of events characterized by a heightened pro-inflammatory response, release of diverse inflammatory cytokines, and activation of the complement system. These processes collectively contribute to an augmented risk of ischemia-reperfusion injury and hemolysis, leading to the destruction of blood cells [15, 56]. In such cases, patients are more prone to pulmonary complications that cause PMV. Nevertheless, many other factors affect CPB time, such as surgeon proficiency and patient condition [57]. The management of CPB time often presents a challenge for doctors and patients, and it is important to improve treatment management, the environment, and patients’ physical and mental conditions.

Finally, we examined the relationship between PMV and in-hospital mortality. We found cardiac surgical patients with PMV had a 14-fold increase in in-hospital mortality compared with that in patients without PMV. Subgroup analyses based on different study populations and PMV definitions revealed that in-hospital mortality significantly increased among PMV patients. Moreover, the relationship between long-term mortality and PMV should be addressed. A large analysis of critically ill patients receiving at least 48 hours of mechanical ventilation showed a 28-day mortality rate of 26.3% [58]. Due to limited evidence, we only analyzed in-hospital mortality. Future studies should explore the effects of PMV on long-term mortality.

We conducted a comprehensive search and rigorous screening of studies for inclusion. NOS was also used to assess the quality. Since the included studies had similar but different clinical settings, we performed subgroup analyses of different study populations and PMV definitions. Given the presence of identical yet distinct clinical settings among the included studies, we conducted subgroup analyses to examine the effects of diverse study populations and variations in PMV definitions. However, our synthesis had some limitations. Firstly, our analysis included only English-language publications, which might have partly caused us to miss relevant non-English publications. Secondly, the number of studies included in each analysis was relatively small. Finally, an exhaustive analysis of other PMV risk factors, such as smoking history and operative time, was not feasible due to their absence or limited coverage in the original studies. Future large-scale clinical trials are required to validate the relationship between those unidentified risk factors and the PMV. In addition, further research should be conducted to determine the weights of these identified risk factors to make them more clinically feasible.

## 5. Conclusions

The incidence of PMV was 20%. Fifteen risk factors were associated with a heightened risk of PMV. However, the potential impacts of higher BMI, heart failure, and arrhythmias require further exploration. Our synthesis also revealed that PMV was significantly associated with increased in-hospital mortality in patients undergoing cardiac surgery. Moreover, we support the definition of PMV as a duration greater than or equal to 24 hours, as it would enable earlier identification of cases with PMV. The findings of our synthesis suggest that the timely identification of risk factors for PMV and prompt recognition and intervention are vital for enhancing patient prognosis. Therefore, future PMV management should better emphasize these identified risk factors.

## Availability of Data and Materials

All data points generated or analyzed during this study are included in this article and there are no further underlying data necessary to reproduce the results.

## Supplementary Material

Supplementary material associated with this article can be found, in the online version, at https://doi.org/10.31083/j.rcm2511409.
