# Supplementary material for: The Incidence, Risk Factors, and Hospital Mortality of Prolonged Mechanical Ventilation among Cardiac Surgery Patients: A Systematic Review and Meta-Analysis
Source: Rev Cardiovasc Med. 2024 Nov 20;25(11):409. doi: 10.31083/j.rcm2511409 (PMC11607491; doi:10.31083/j.rcm2511409)
Supplement: Supplementary file 1 [file 2153-8174-25-11-409-s1.zip › Supplementary material 2.docx]

**Search strategies**

**1. PubMed search strategy**

#1: Cardiac Surgical Procedures [mh]

#2: Cardiac Surgical Procedures or Procedure, Cardiac Surgical or Procedures or Procedures, Heart or Cardiac Surgical Procedure or Heart Surgical Procedures or Procedure, Heart Surgical or Procedures, Heart Surgical or Surgical Procedure, Heart or Heart Surgical Procedure or cardiopulmonary bypass or extracorporeal circulation

#3: #1or #2

#4:[Thoracic Surgery](https://www.ncbi.nlm.nih.gov/mesh/68013903), [Thoracic Surgical Procedures](https://www.ncbi.nlm.nih.gov/mesh/68019616) or Procedure

#5: Coronary artery bypass grafting or Valve surgery or Aortic surgery

#6: #4 or #5

#7: #3 or #6

#8: Prolonged mechanical ventilation or Delayed extubation or Extubation failure

#9: #7 and #8

**631 Search results**

Results obtained August 31, 2021.

**2. Cochrance Library search strategy**

#1:“Cardiac Surgical Procedures” OR “Cardiac Surgical Procedure” OR “Cardiac Surgical” OR “Cardiac Procedures” OR “Cardiac Procedure” OR “Heart Surgical Procedure” OR “Heart Surgical Procedures” OR “Heart Surgical” OR “Heart Procedures” OR “Cardiopulmonary bypass” OR “Extracorporeal circulation”

#2: (“Coronary artery bypass grafting”):ti,ab,kw or (“Valve surgery”):ti,ab,kw or (“Aortic surgery”):ti,ab,kw

#3: #1 and #2

#4: (“prolonged mechanical ventilation”):ti,ab,kw OR (“delayed extubation”):ti,ab,kw OR (“extubation failure”):ti,ab,kw

#5: #3 and #4

**52 Search results**

Results obtained August 31, 2021.

**3. Embase search strategy**

#1: “heart surgery”/exp

#2: “heart surgery” or “cardiac surgery” or “cardiac surgical procedures” or “cardiosurgery” or “heart operation” or “open heart surgery” or “extracorporeal circulation” or “cardiopulmonary bypass”

#3: #1 or #2

#4: “Prolonged mechanical ventilation” or “Delayed extubation” or “Extubation failure”

#5: #3 and #4

**877 Search results**

Results obtained August 31, 2021.

**4. Web of Science**

#1 TS= (“Cardiac Surgical Procedures” OR “Cardiac Surgical Procedure” OR “Cardiac Surgical” OR “Cardiac Procedures” OR “Cardiac Procedure” OR “Heart Surgical Procedure” OR “Heart Surgical Procedures” OR “Heart Surgical” OR “Heart Procedures” OR “Cardiopulmonary bypass” OR “Extracorporeal circulation”)

#2 TS= (“[Thoracic Surgery](https://www.ncbi.nlm.nih.gov/mesh/68013903)” OR “[Thoracic Surgical Procedures](https://www.ncbi.nlm.nih.gov/mesh/68019616)” OR “Thoracic Surgical Procedure”)

#3 TS= (“Coronary artery bypass grafting” or “Valve surgery” or “Aortic surgery”)

#4 OR/1-3

#5 TS= (“prolonged mechanical ventilation” OR “delayed extubation” OR “extubation failure”)

#6 #4 and #5

**203 Search results**

Results obtained August 31, 2021.
