# Supplementary material for: The Incidence, Risk Factors, and Hospital Mortality of Prolonged Mechanical Ventilation among Cardiac Surgery Patients: A Systematic Review and Meta-Analysis
Source: Rev Cardiovasc Med. 2024 Nov 20;25(11):409. doi: 10.31083/j.rcm2511409 (PMC11607491; doi:10.31083/j.rcm2511409)
Supplement: Supplementary file 1 [file 2153-8174-25-11-409-s1.zip › Supplementary material 3.docx]

**Table 1. The Critical Appraisal of Included Studies Using Newcastle-Ottawa Scale (NOS).**

| **Study** | **Score by NOS Category** | | | |
| --- | --- | --- | --- | --- |
|  | **Selection** | **Comparability** | **Outcome/Exposure** | **Overall Score** |
| Engle J et al.1999 | 3 | 1 | 2 | 6 |
| Kern H et al.2001 | 3 | 1 | 3 | 7 |
| Légaré Jf et al.2001 | 3 | 1 | 2 | 6 |
| Yende S et al.2004 | 3 | 1 | 3 | 7 |
| Natarajan K et al.2006 | 3 | 1 | 2 | 6 |
| Lei Q et al.2009 | 3 | 1 | 3 | 7 |
| Shirzad M et al.2010 | 3 | 1 | 2 | 6 |
| Christian K et al.2011 | 3 | 1 | 2 | 6 |
| Piotto RF et al.2012 | 3 | 1 | 2 | 6 |
| Siddiqui MM et al.2012 | 3 | 1 | 2 | 6 |
| Saleh HZ et al.2012 | 3 | 1 | 3 | 7 |
| Bartz RR et al.2015 | 3 | 2 | 3 | 8 |
| Gumus F et al.2015 | 3 | 1 | 2 | 6 |
| Sharma V et al.2017 | 3 | 1 | 3 | 7 |
| Wise ES et al.2017 | 3 | 1 | 2 | 6 |
| Chen YJ et al.2019 | 3 | 1 | 2 | 6 |
| Hsu H et al.2019 | 3 | 1 | 3 | 7 |
| Papathanasiou M et al. 2019 | 3 | 1 | 3 | 7 |
| Aksoy R et al.2021 | 3 | 1 | 3 | 7 |
| Ge M et al.2021 | 3 | 1 | 3 | 7 |
| Kreibich M et al.2022 | 3 | 1 | 3 | 7 |
| Lin L et al.2022 | 3 | 1 | 3 | 7 |
| Li X et al.2022 | 3 | 2 | 3 | 8 |
| Michaud L et al.2022 | 3 | 1 | 2 | 6 |
| Meng Y et al.2022 | 3 | 2 | 3 | 8 |
| Sankar A et al.2022 | 3 | 2 | 2 | 7 |
| Xie Q et al.2022 | 3 | 1 | 3 | 7 |
| Xiao Y et al.2022 | 3 | 1 | 3 | 7 |
| Zhang Q et al.2022 | 3 | 1 | 3 | 7 |
| Xiao S et al. 2022 | 3 | 1 | 3 | 7 |
| Shahram R et al.2023 | 3 | 1 | 3 | 7 |
| Yuankai Z et al.2023 | 2 | 1 | 3 | 6 |

**Table 2 Sensitivity analysis**

| **Out-come** | **Before sensitivity analysis** | | | **Remove study** | **After sensitivity analysis** | | |
| --- | --- | --- | --- | --- | --- | --- | --- |
|  | **Effect estimate** | ***P*** | ***I*^2^ (%)** |  | **Effect estimate** | ***P*** | ***I*^2^ (%)** |
| **Hyper-tension** | 1.17  (0.91, 1.50) | 0.23 | 75.5 | Kreibich M et al.2022 | 1.28  (1.02, 1.61) | 0.03 | 60.5 |
| **Diabetes** | 1.31  (1.00,1.72) | 0.09 | 76.9 | Papathanasiou M et al. 2019 | 1.42  (1.11,1.81) | <0.01 | 73.2 |

**Table 3 Level of evidence assessment of meta-analysis results using Grading of Recommendations, Assessment, Development and Evaluations (GRADE).**

| Variable | GRADE Certainty Rating |
| --- | --- |
| **Pre-Operative** | |
| Advanced age (years) | ⨁⨁⨁⨁High |
| Being female | ⨁⨁⨁⨁High |
| EF<50 | ⨁⨁⨁◯Moderate |
| BMI (kg/m^2^) | ⨁⨁◯◯Low |
| BMI>28(kg/m^2^) | ⨁⨁⨁◯Moderate |
| NYHA class≥ Ⅲ | ⨁⨁⨁◯Moderate |
| Hypertension | ⨁⨁◯◯Low |
| Diabetes | ⨁⨁◯◯Low |
| Three or more vessel disease | ⨁⨁◯◯Low |
| COPD | ⨁⨁⨁⨁High |
| Chronic renal failure | ⨁⨁⨁⨁High |
| Heart failure | ⨁⨁◯◯Low |
| Arrhythmology | ⨁⨁◯◯Low |
| Emergency operation | ⨁⨁◯◯Low |
| Previous cardiac surgery | ⨁⨁⨁⨁High |
| Perioperaitive MI | ⨁⨁◯◯Low |
| Creatinine | ⨁⨁◯◯Low |
| Higher WBC count | ⨁⨁⨁⨁High |
| **Intra-Operative** | |
| CPB (min) | ⨁⨁◯◯Low |
| CPB>120min | ⨁⨁⨁◯Moderate |
| Aortic cross-clamp time (min) | ⨁⨁◯◯Low |
