# Supplementary material for: The Incidence, Risk Factors, and Hospital Mortality of Prolonged Mechanical Ventilation among Cardiac Surgery Patients: A Systematic Review and Meta-Analysis
Source: Rev Cardiovasc Med. 2024 Nov 20;25(11):409. doi: 10.31083/j.rcm2511409 (PMC11607491; doi:10.31083/j.rcm2511409)
Supplement: Supplementary file 1 [file 2153-8174-25-11-409-s1.zip › Supplementary material 4.docx]

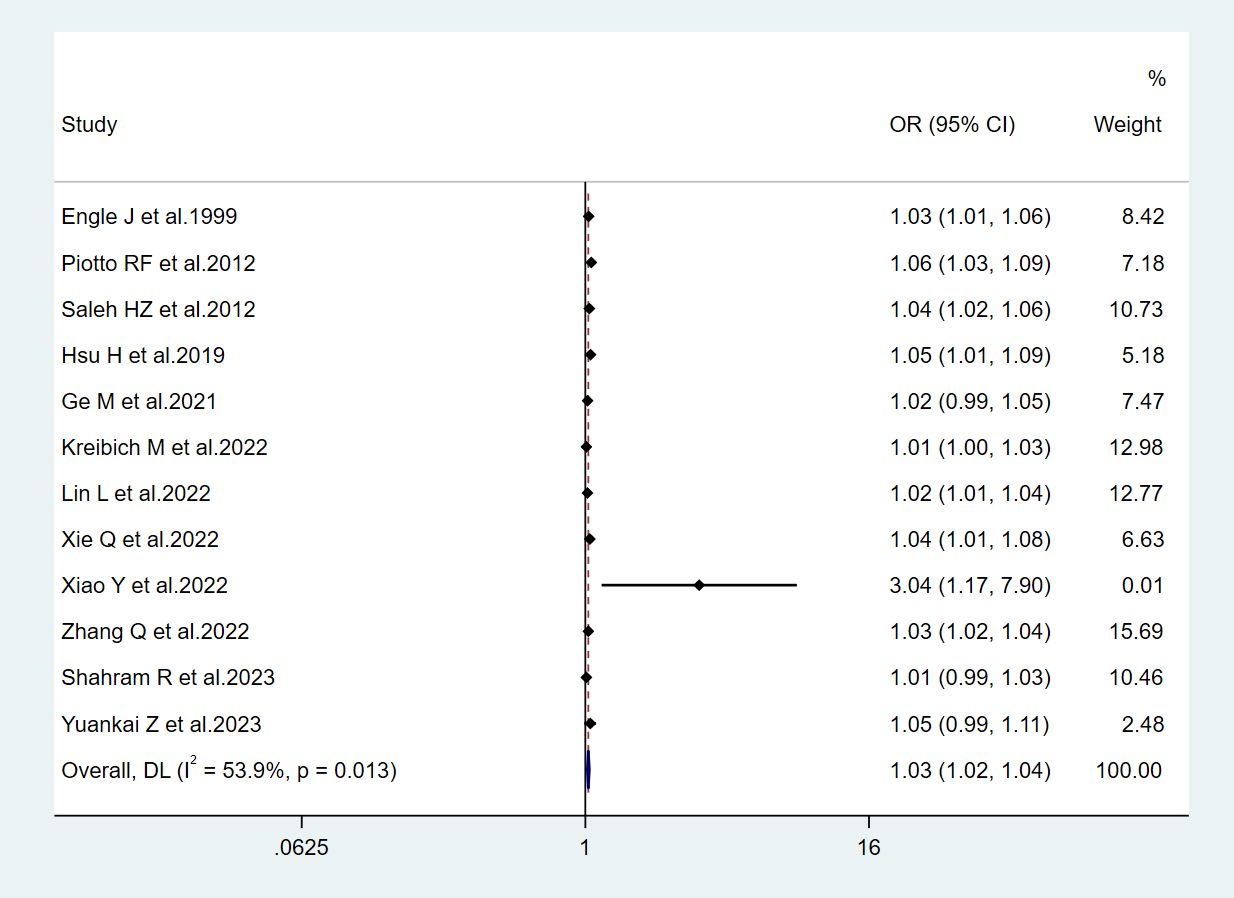


1.1 The forest plot of advanced aged.


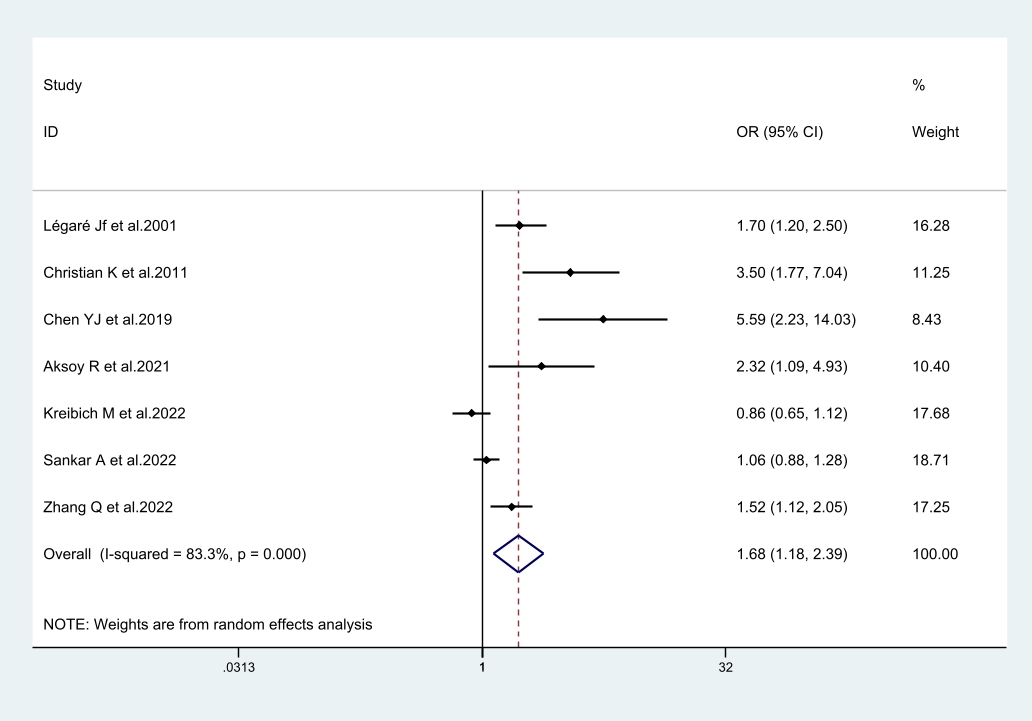


1.2 The forest plot of being female.


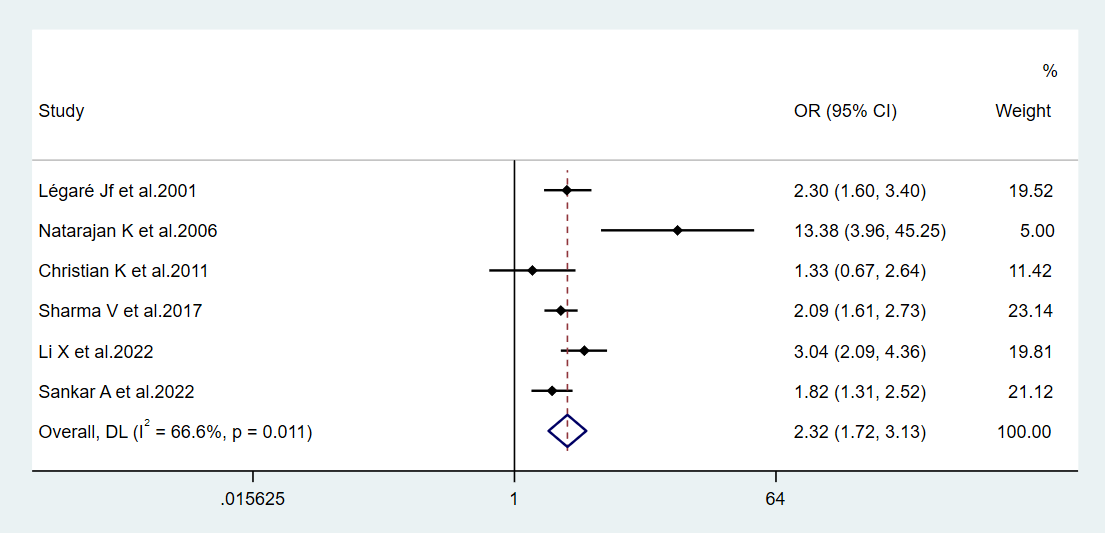


1.3 The forest plot of ejection fraction<50.


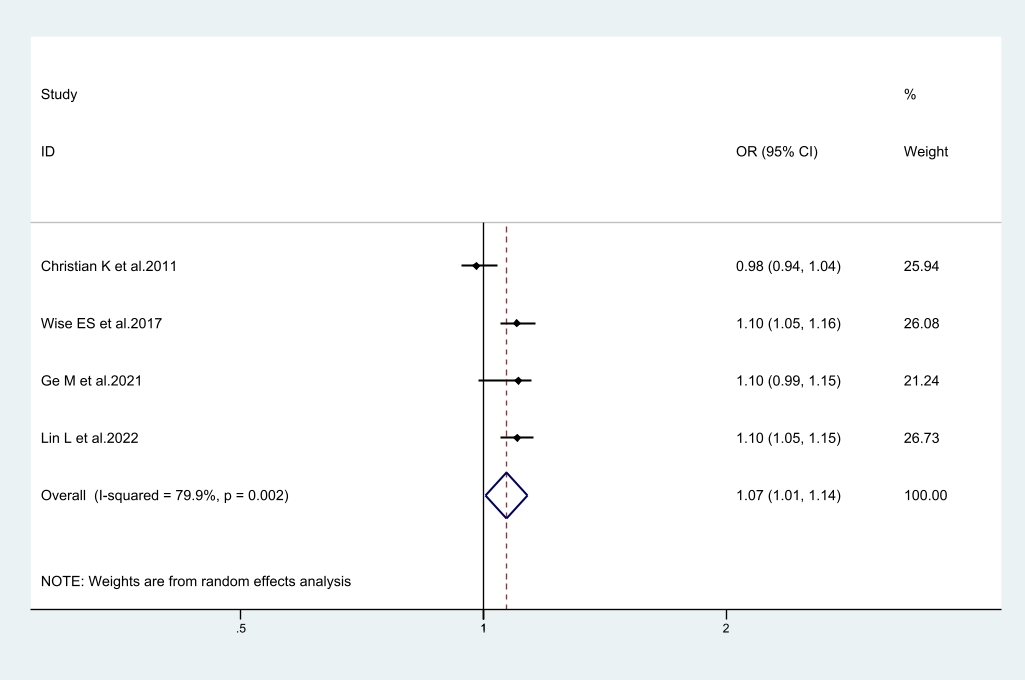


1.4 The forest plot of body mass index.


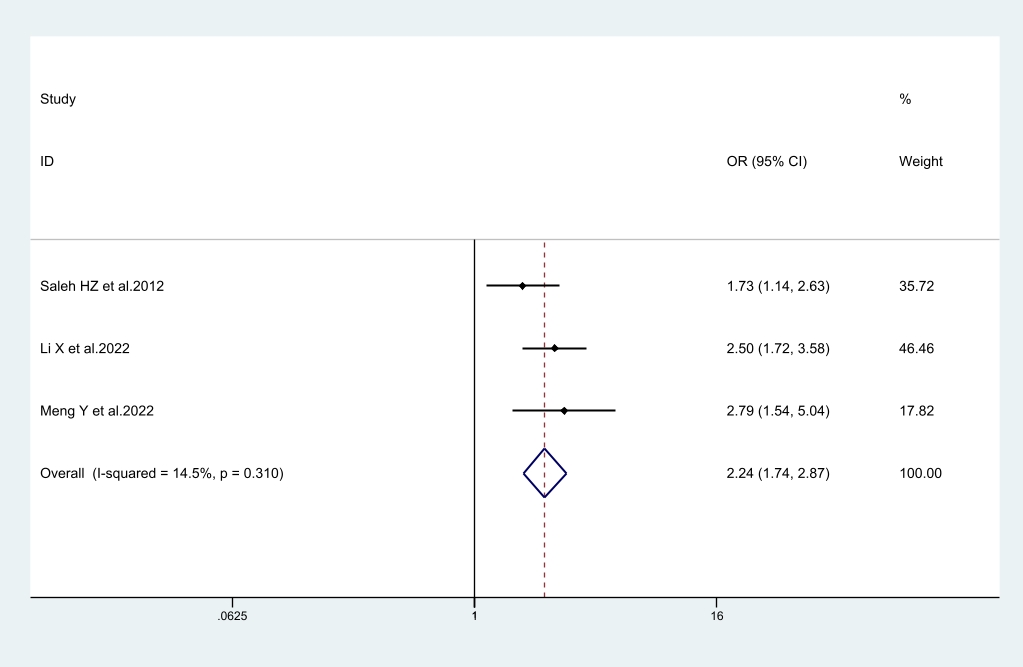


1.5 The forest plot of body mass index > 28.


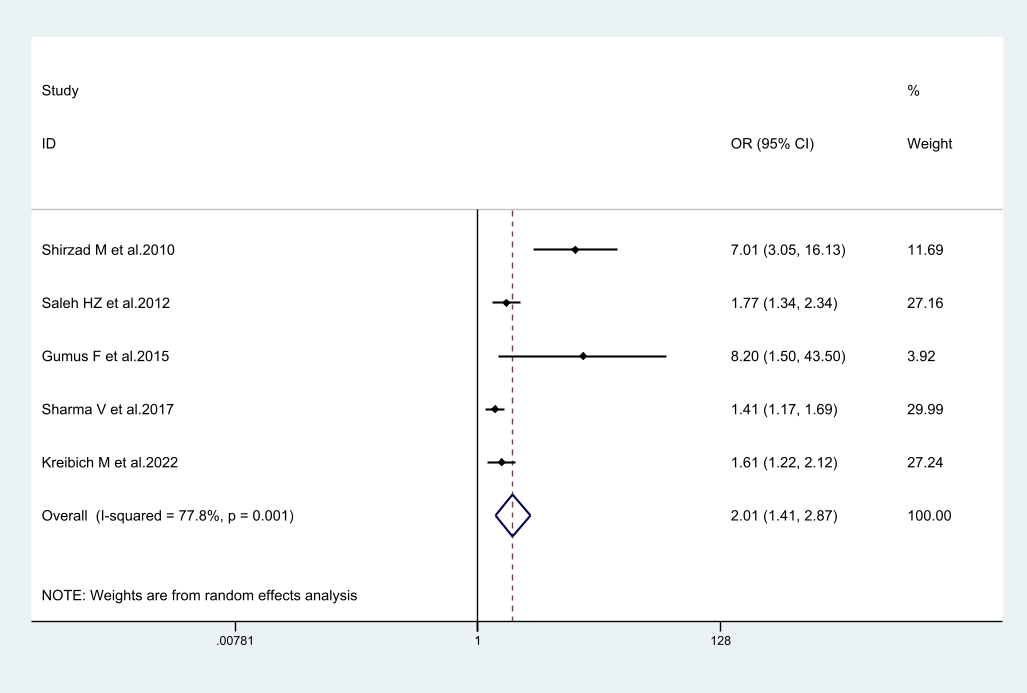


1.6 The forest plot of New York heart association class ≥ Ⅲ.


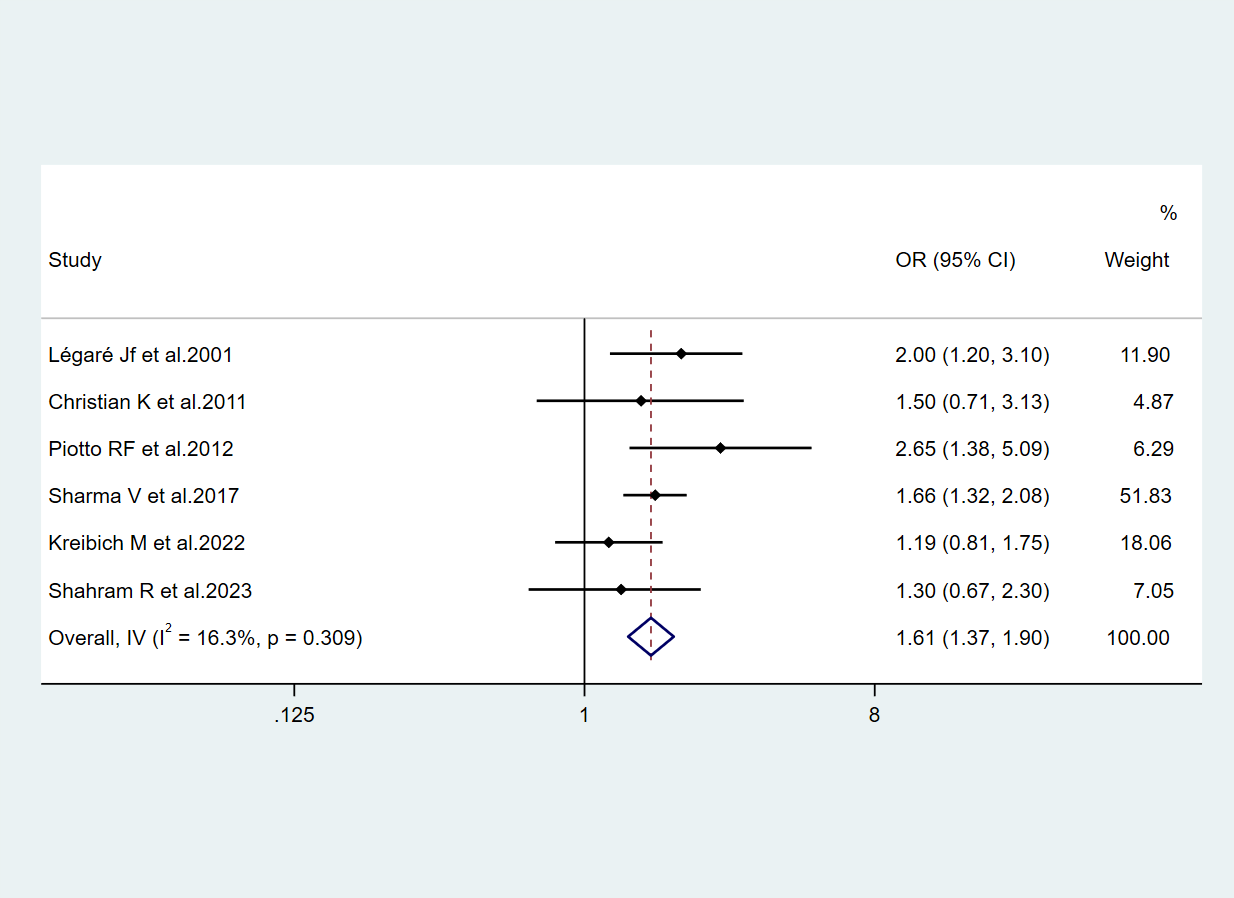


1.7 The forest plot of chronic obstructive pulmonary disease.

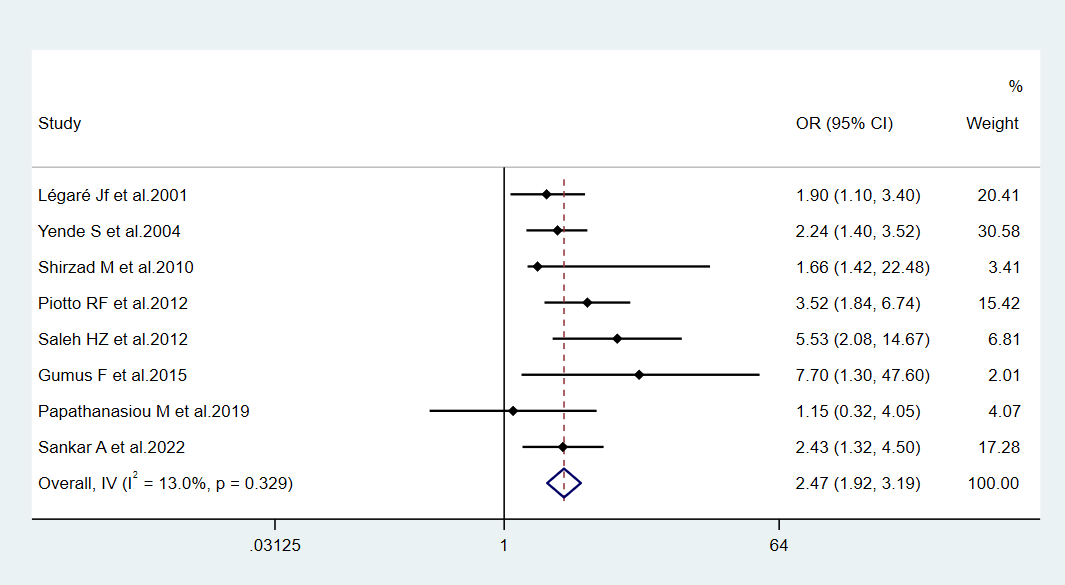


1.8 The forest plot of chronic renal failure.


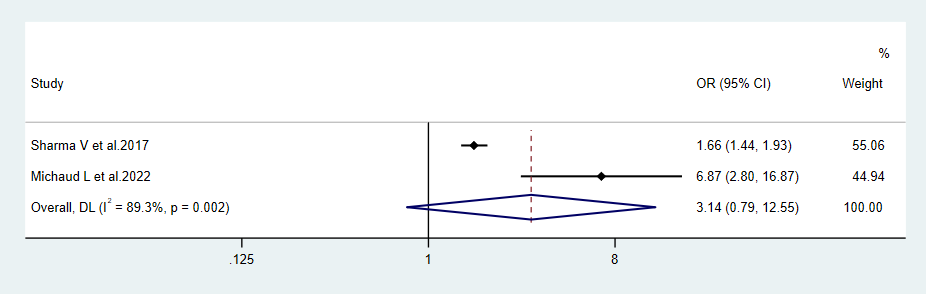


1.9 The forest plot of heart failure.


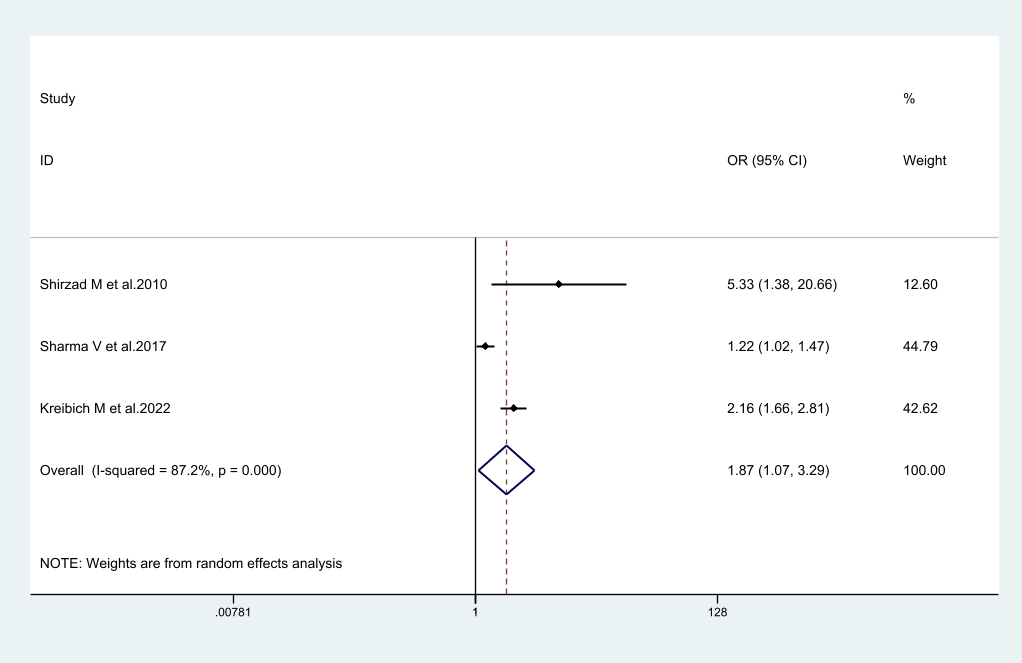


1.10 The forest plot of arrhythmia.


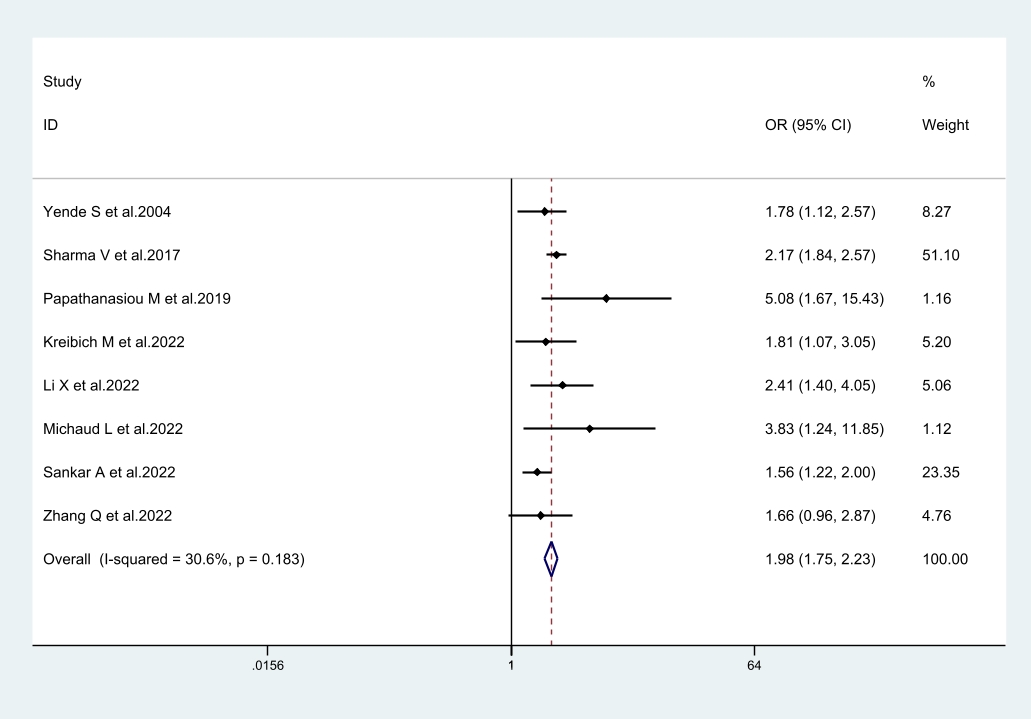


1.11 The forest plot of previous cardiac surgery.


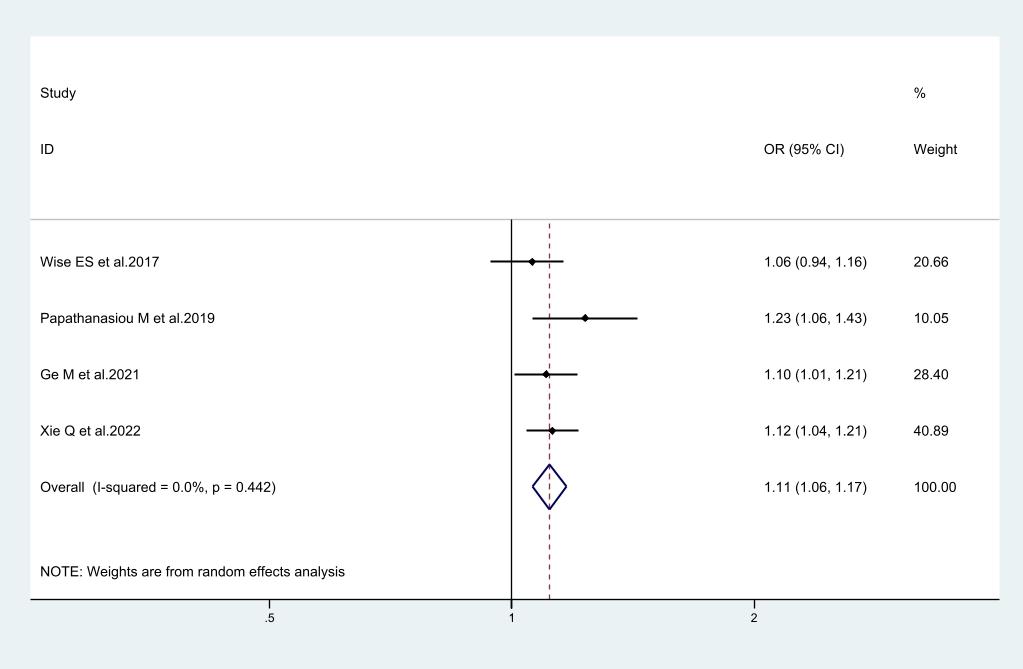


1.12 The forest plot of higher white blood cell count.

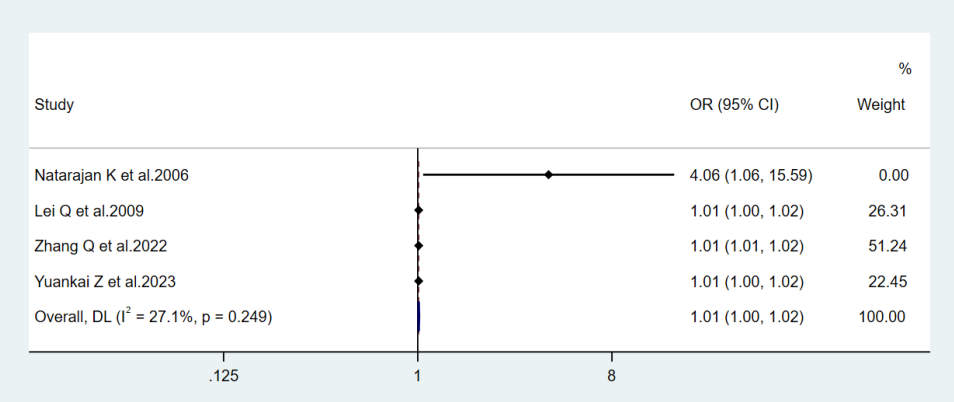


1.13 The forest plot of creatinine.
**Fig.1 Prolonged mechanical ventilation pre-operative risk factor forest plots.**


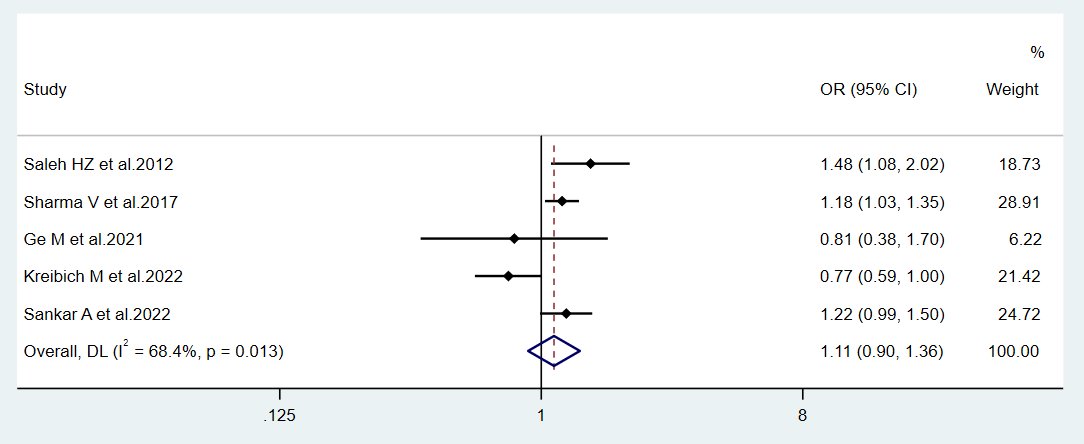


2.1 The forest plot of hypertension.


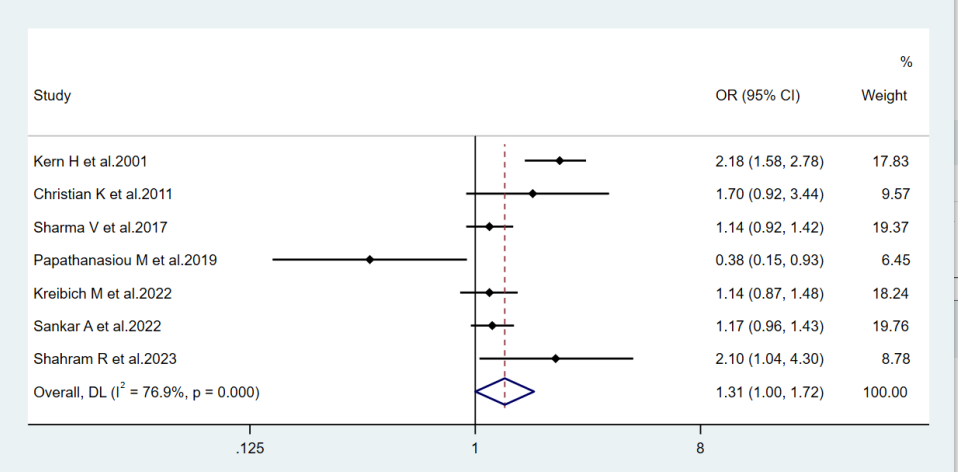


2.2 The forest plot of diabetes.


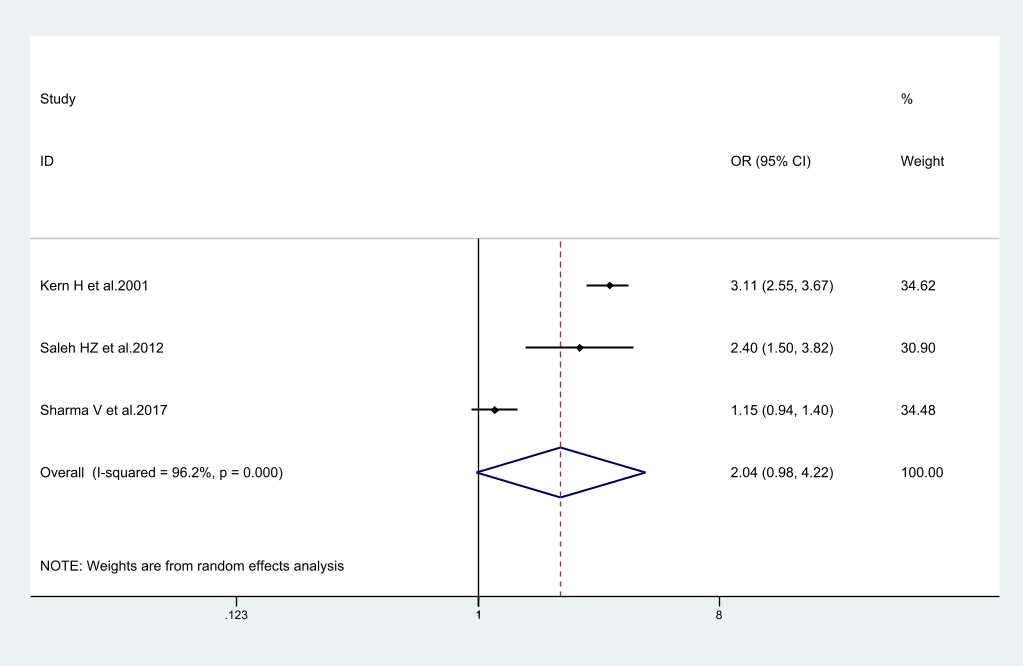


2.3 The forest plot of three or more vessel disease.


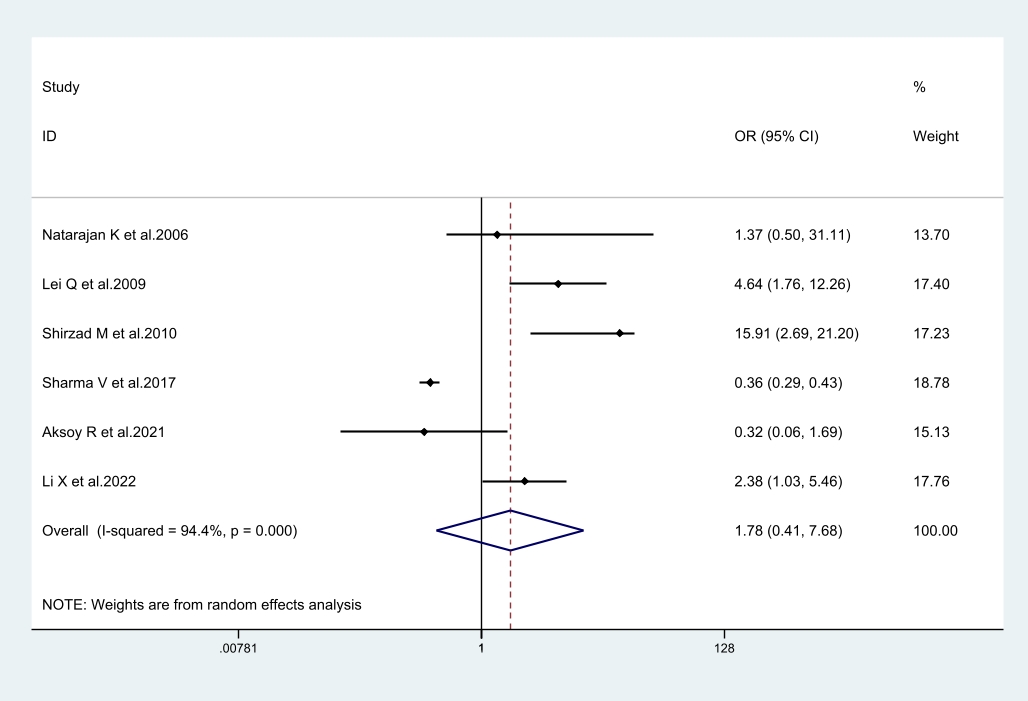


2.4 The forest plot of emergency surgery.


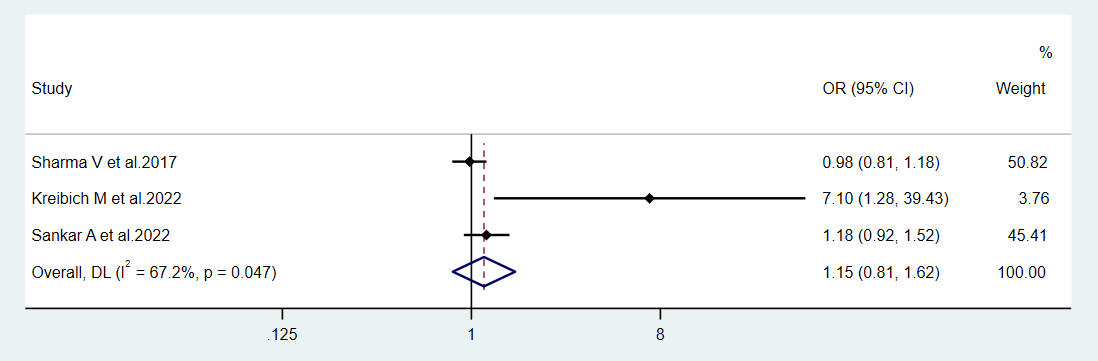


2.5 The forest plot of perioperaitive stroke.

**Fig.2 Prolonged mechanical ventilation pre-operative non-risk factor forest plot.**


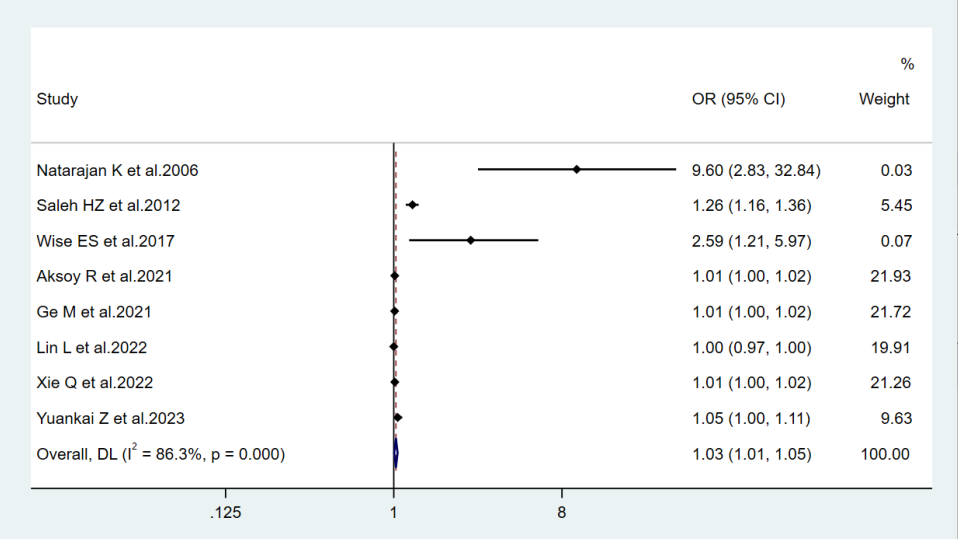


3.1 The forest plot of longer cardiopulmonary bypass time.


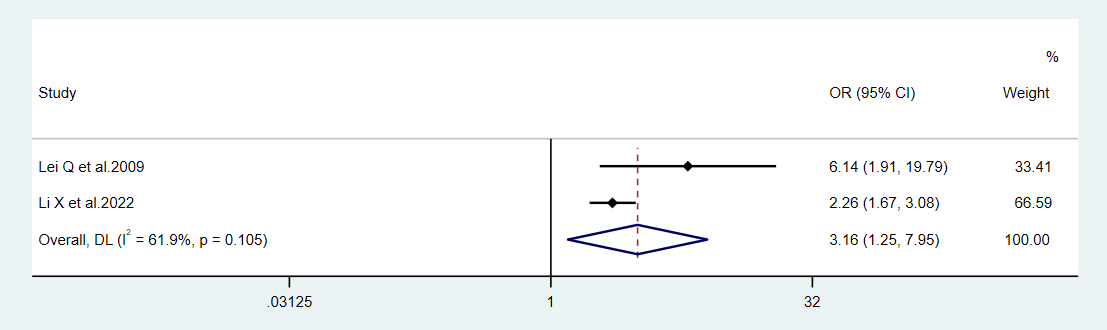


3.2 The forest plot of cardiopulmonary bypass time > 120min.


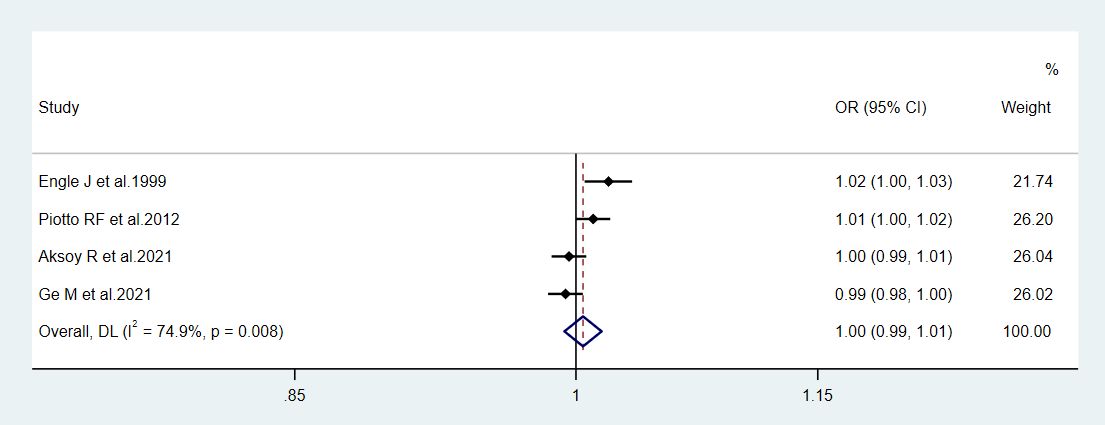


3.3 The forest plot of cross-clamp time.

**Fig.3 Prolonged mechanical ventilation intraoperative risk factor forest plot.**


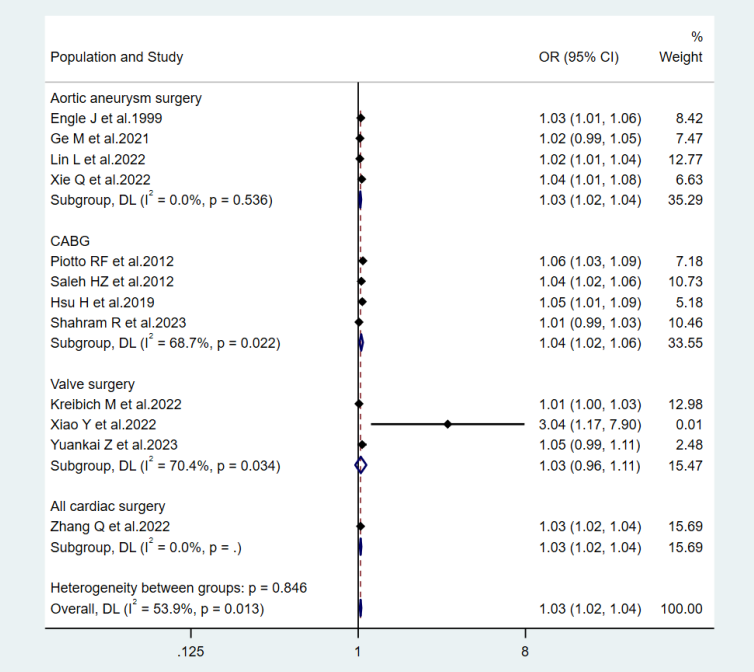


4.1 The subgroup analysis of advanced age.


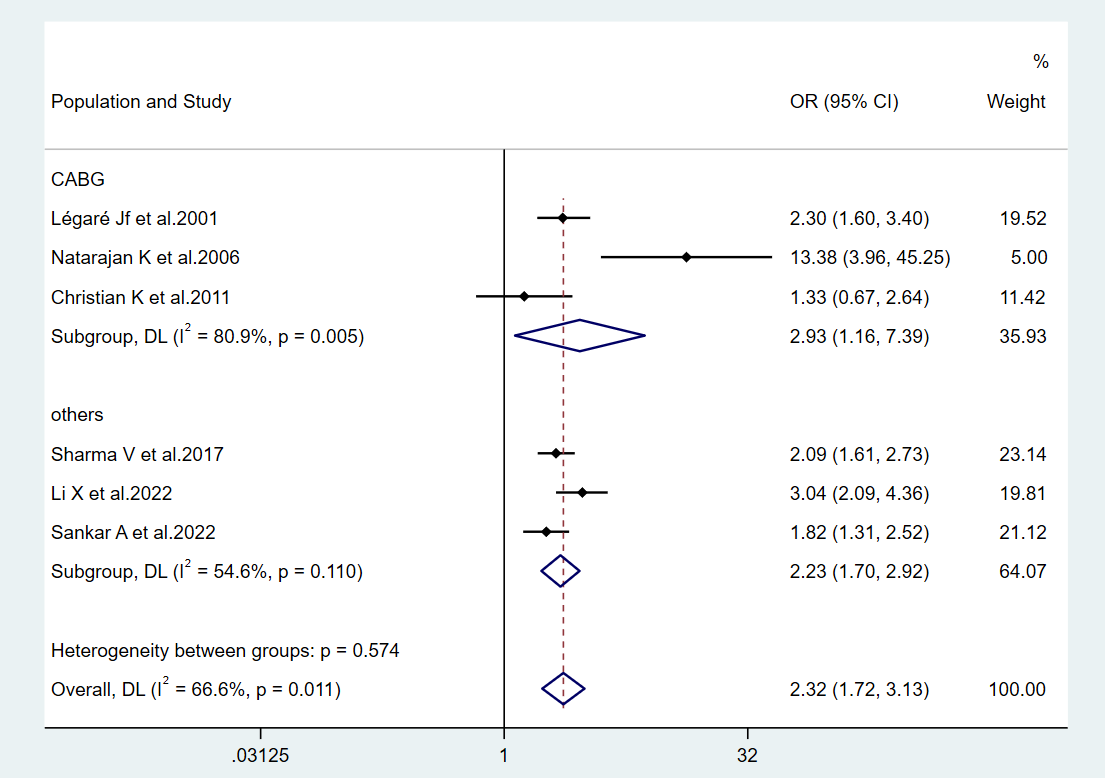


4.2 The subgroup analysis of ejection fraction < 50.


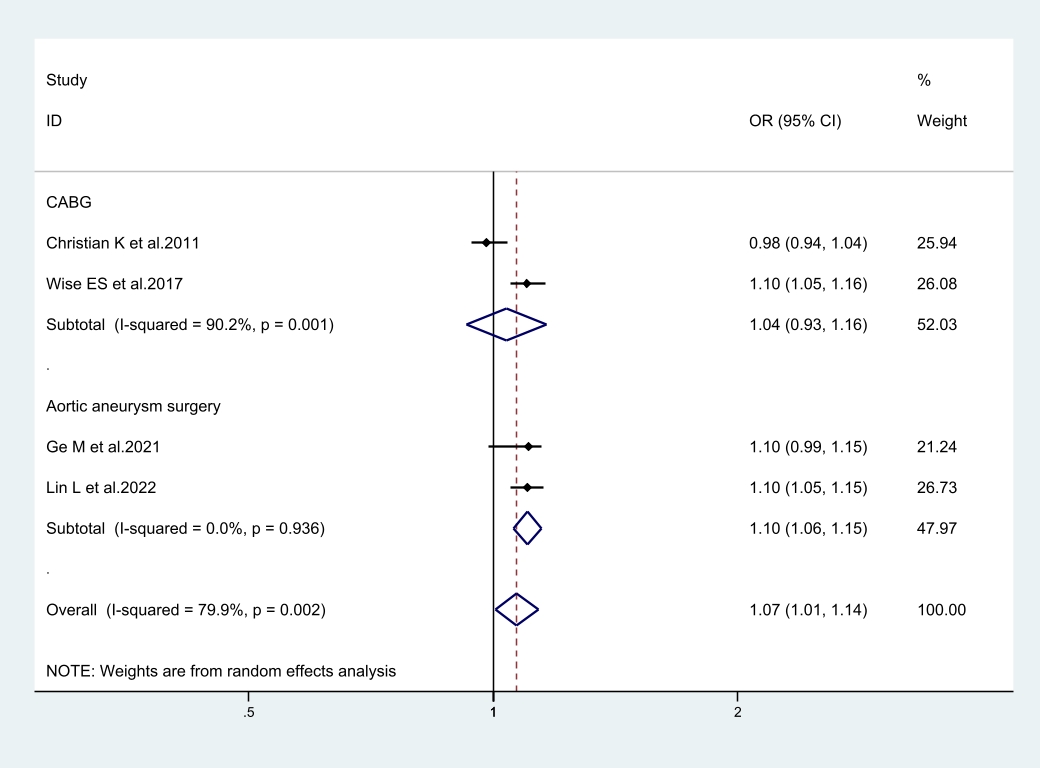


4.3 The subgroup analysis of higher body mass index.


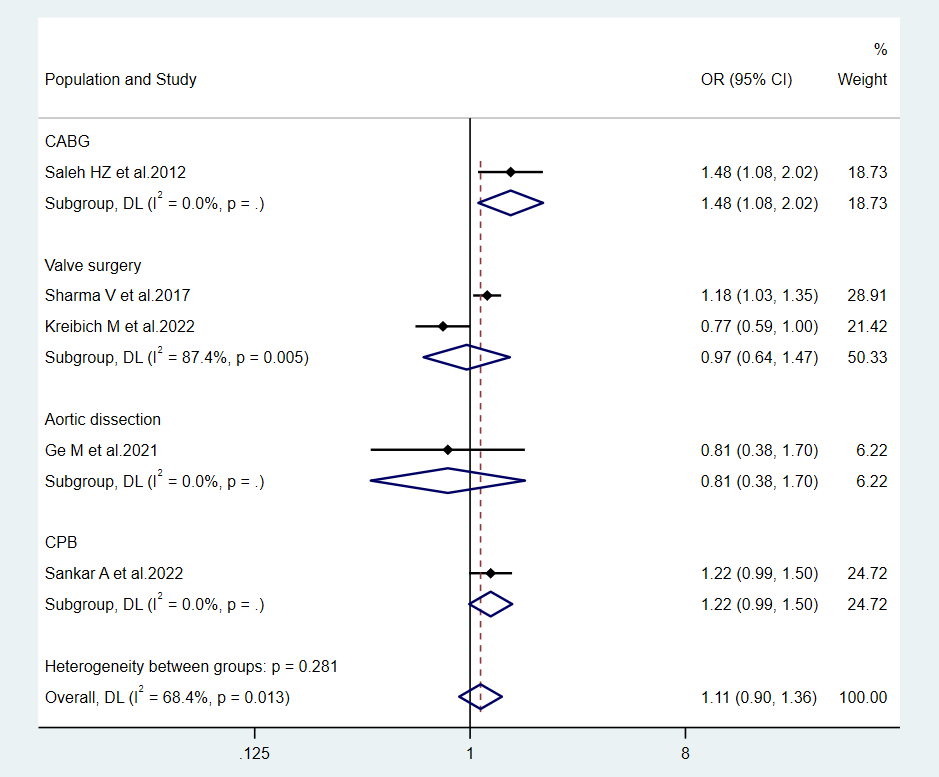


4.4 The subgroup analysis of hypertension.


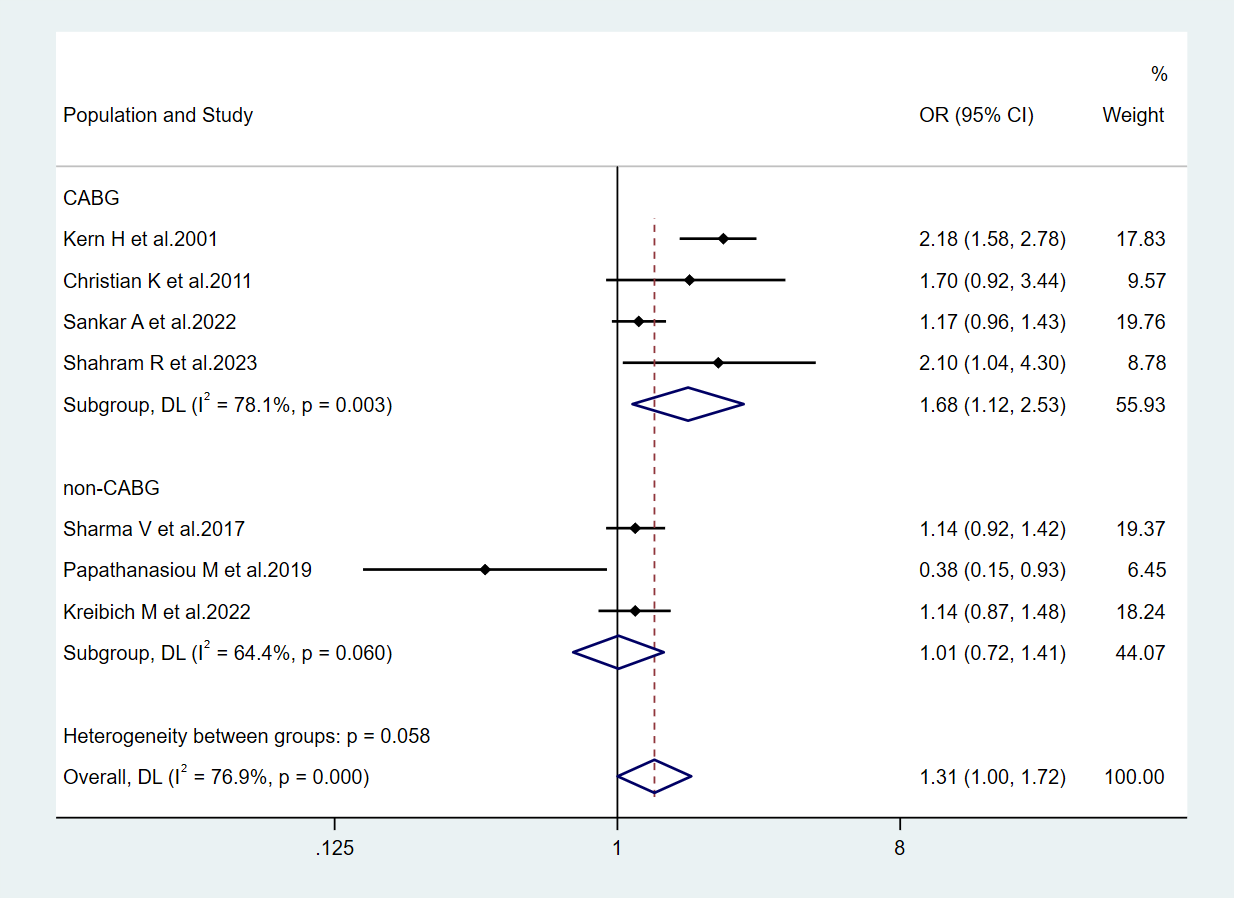


4.5 The subgroup analysis of diabetes.


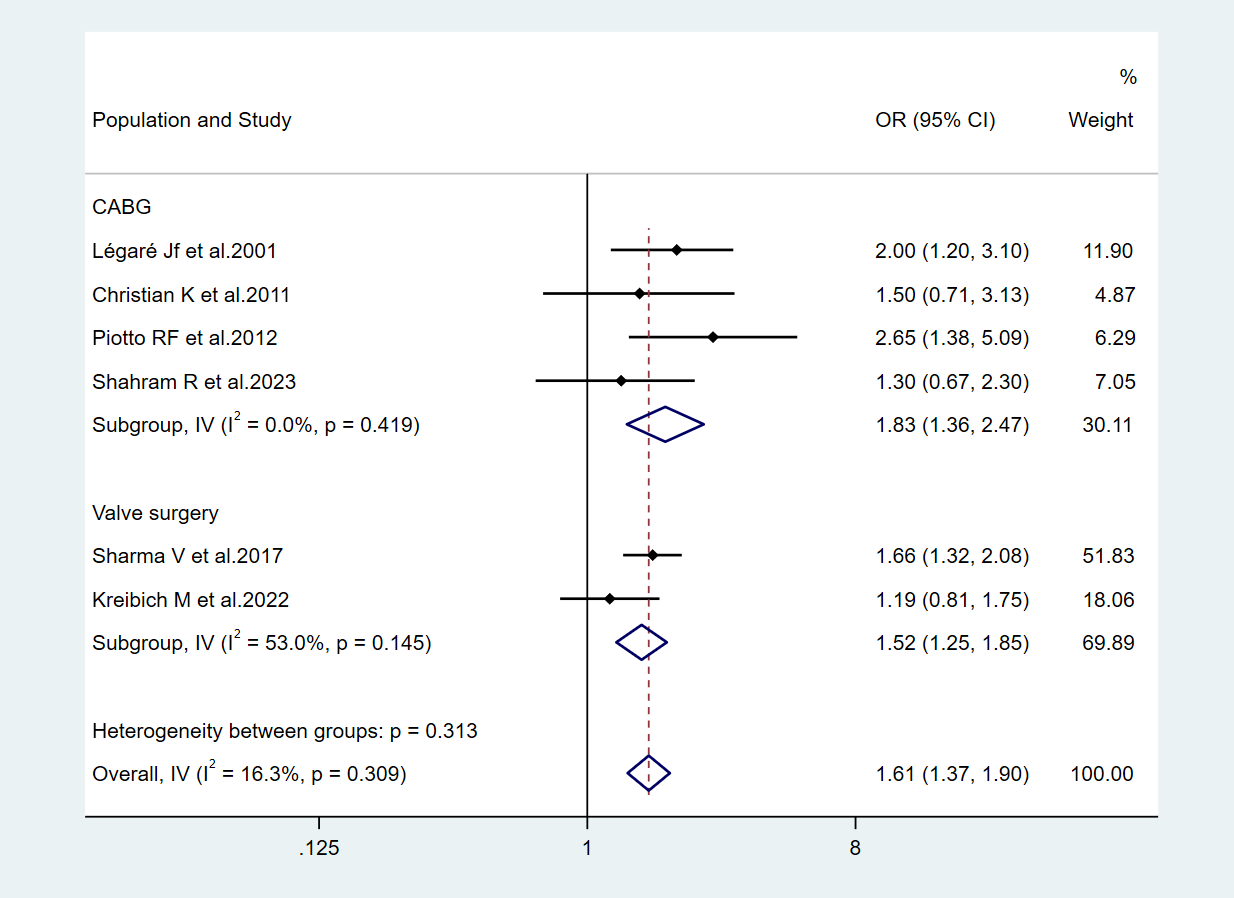


4.6 The subgroup analysis of chronic obstructive pulmonary disease.


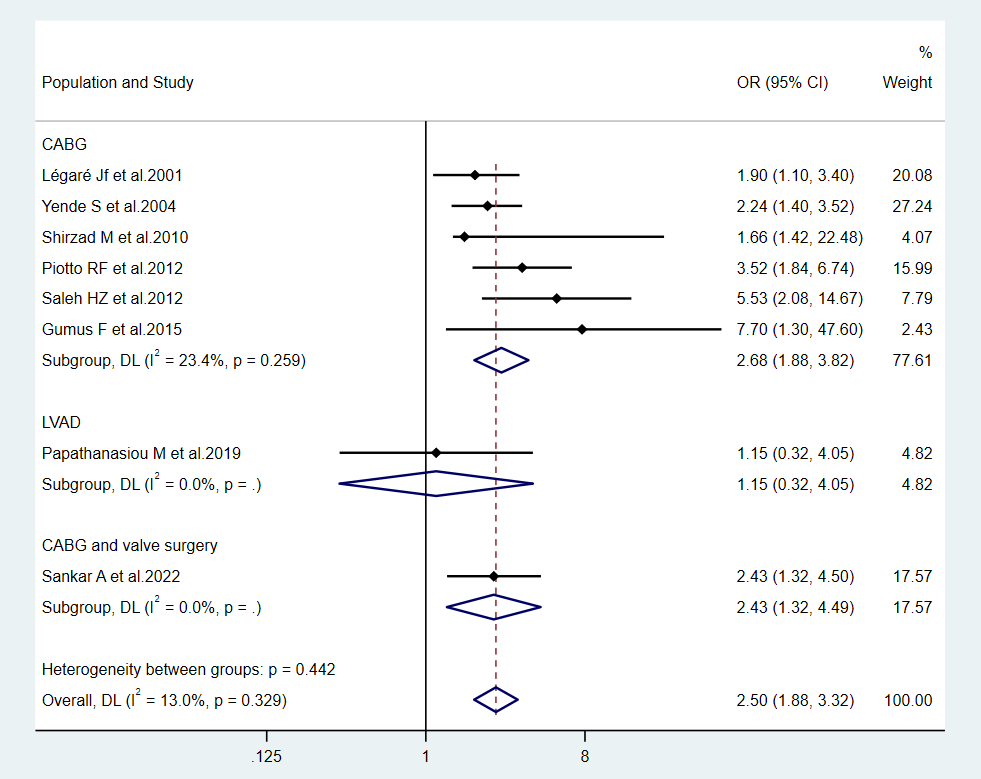


4.7 The subgroup analysis of chronic renal failure.


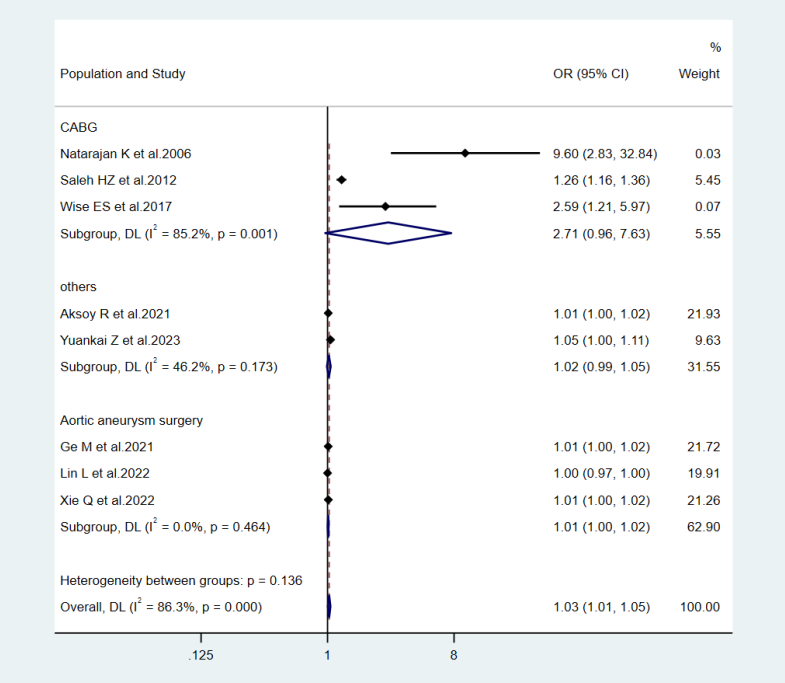


4.8 The subgroup analysis of longer cardiopulmonary bypass time.

**Fig.4 Subgroup analysis of the study population.**


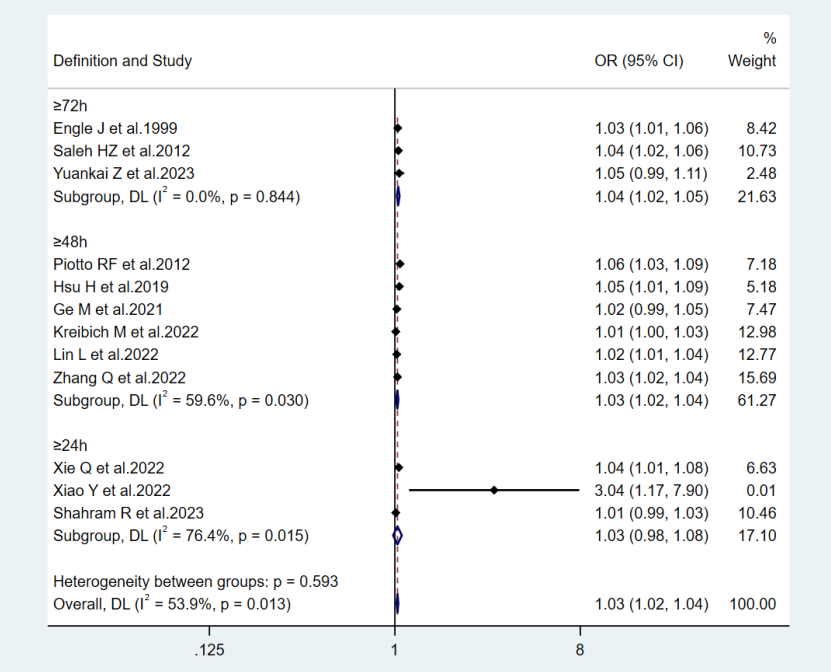


5.1 The subgroup analysis of advanced age.


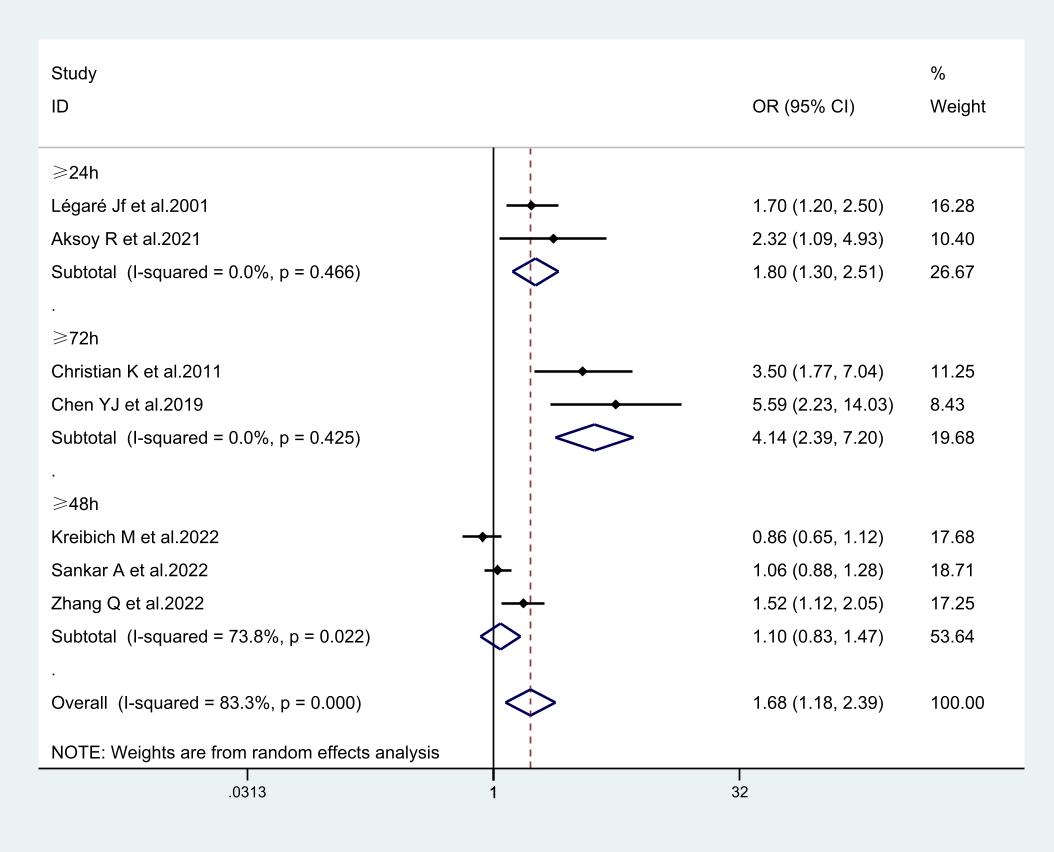


5.2 The subgroup analysis of being female.


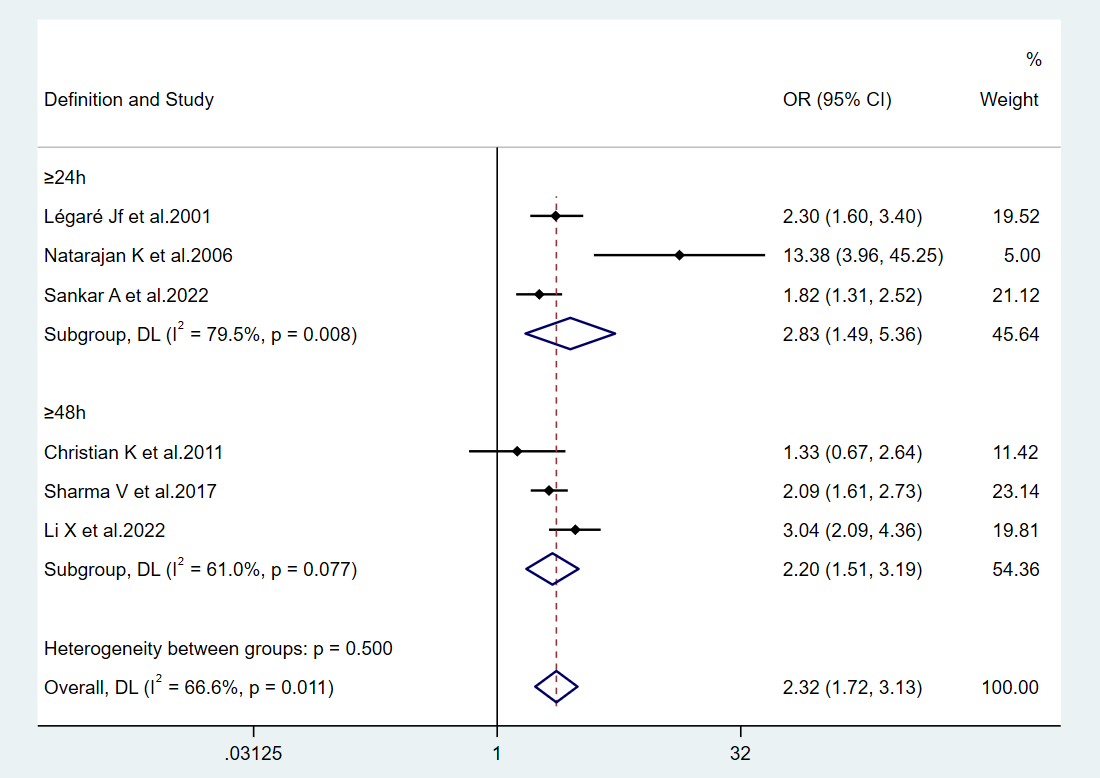


5.3 The subgroup analysis of ejection fraction < 50.


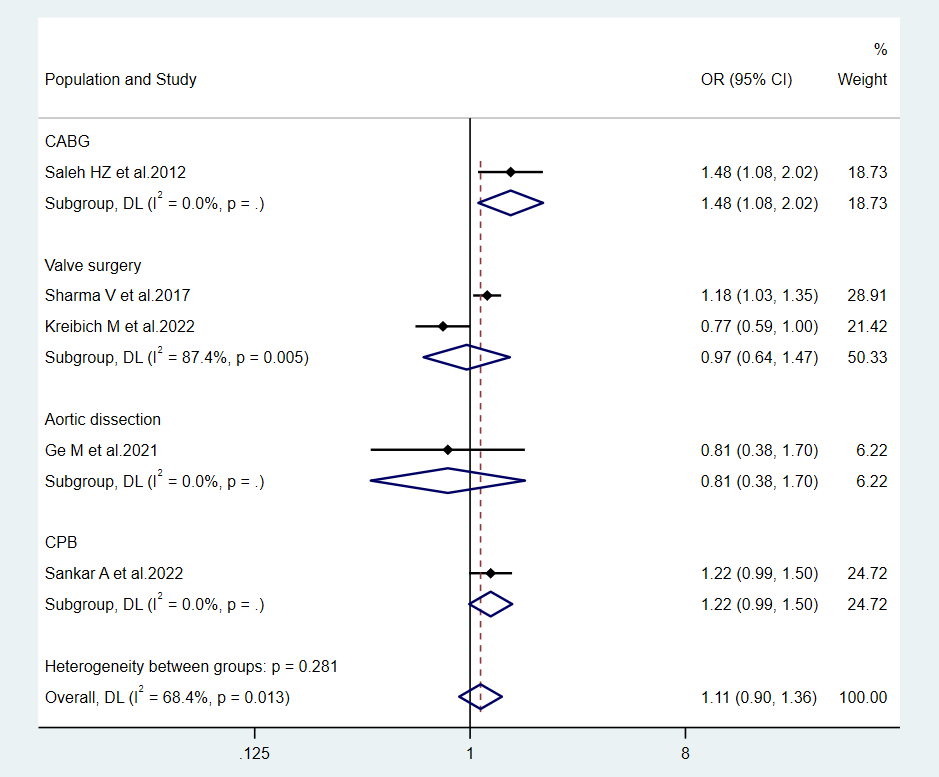


5.4 The subgroup analysis of hypertension.


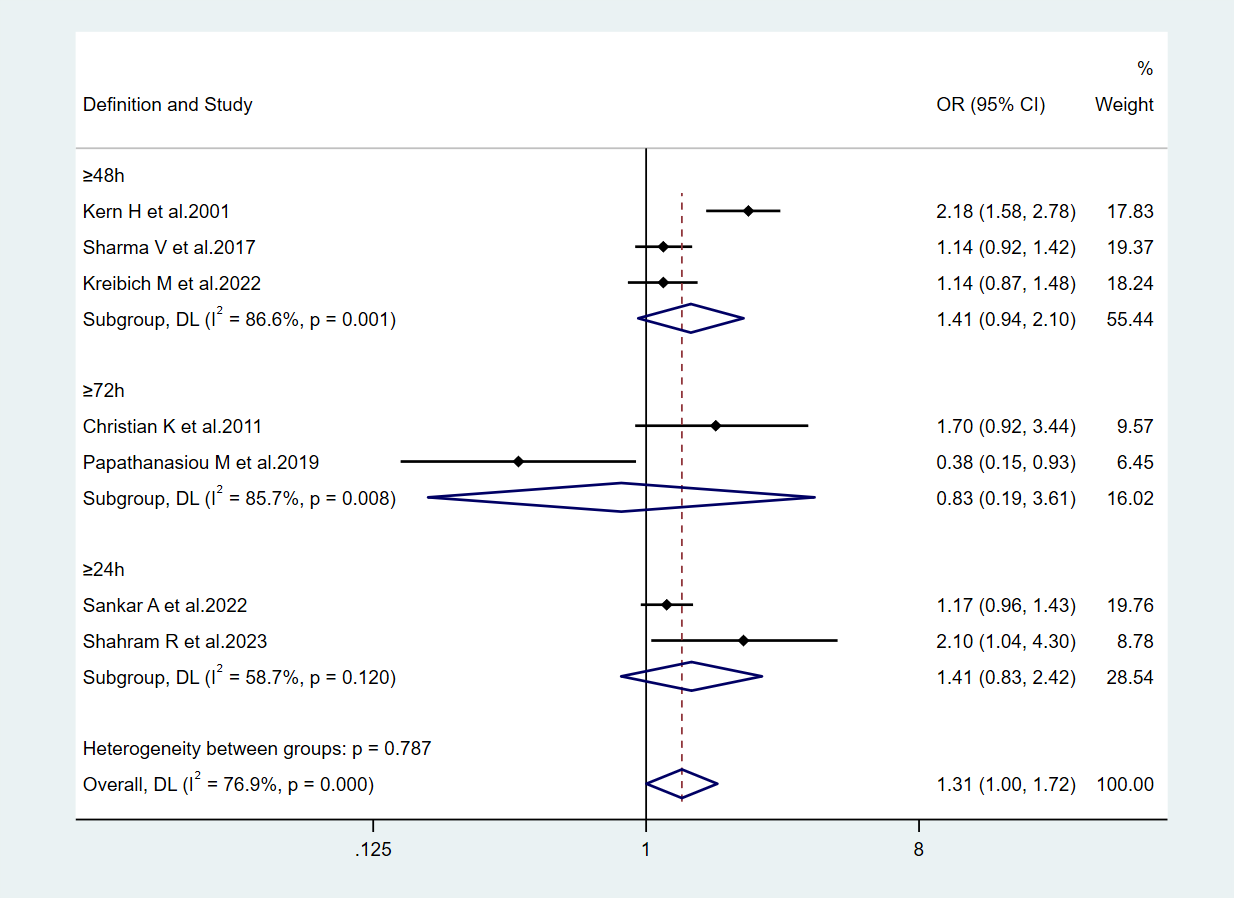


5.5 The subgroup analysis of diabetes.

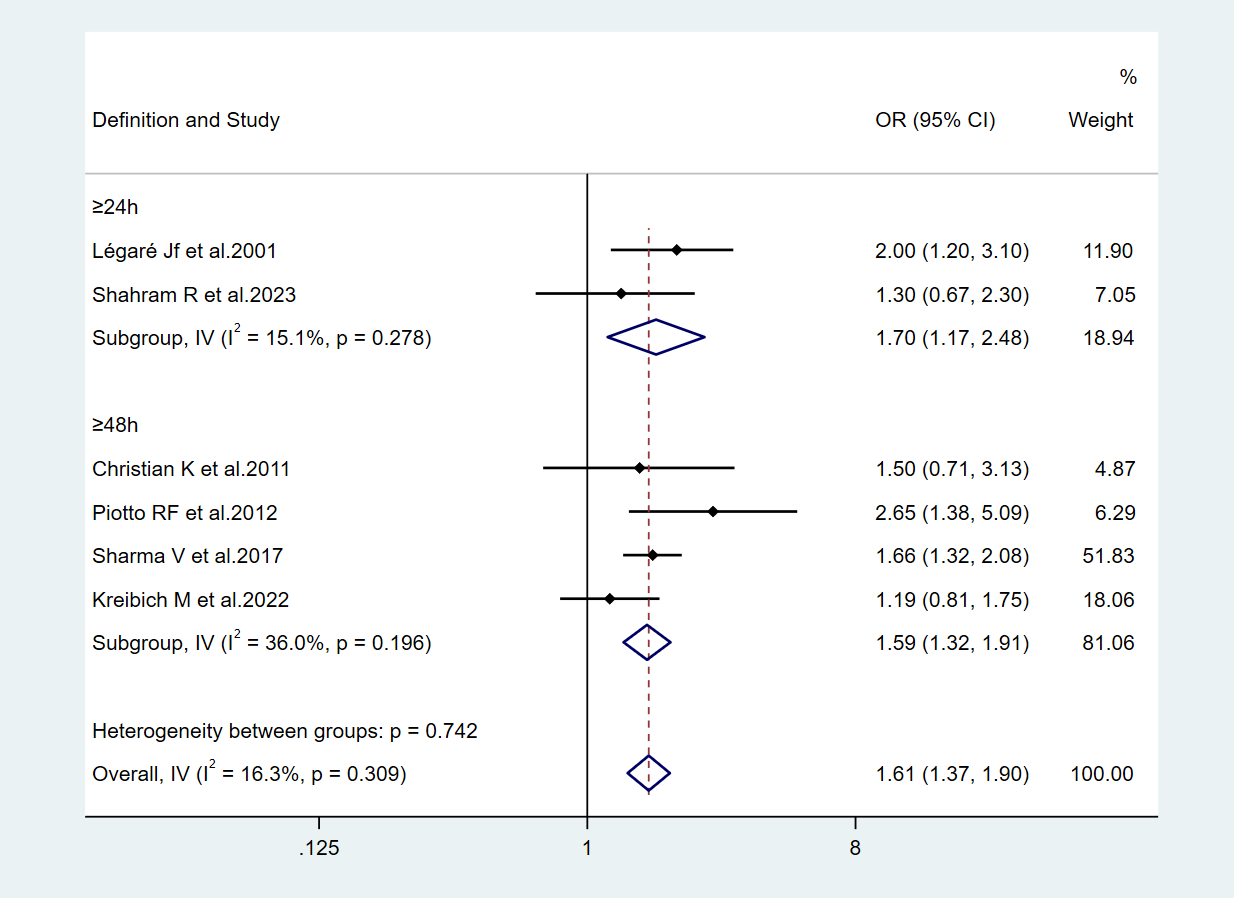


5.6 The subgroup analysis of chronic obstructive pulmonary disease.


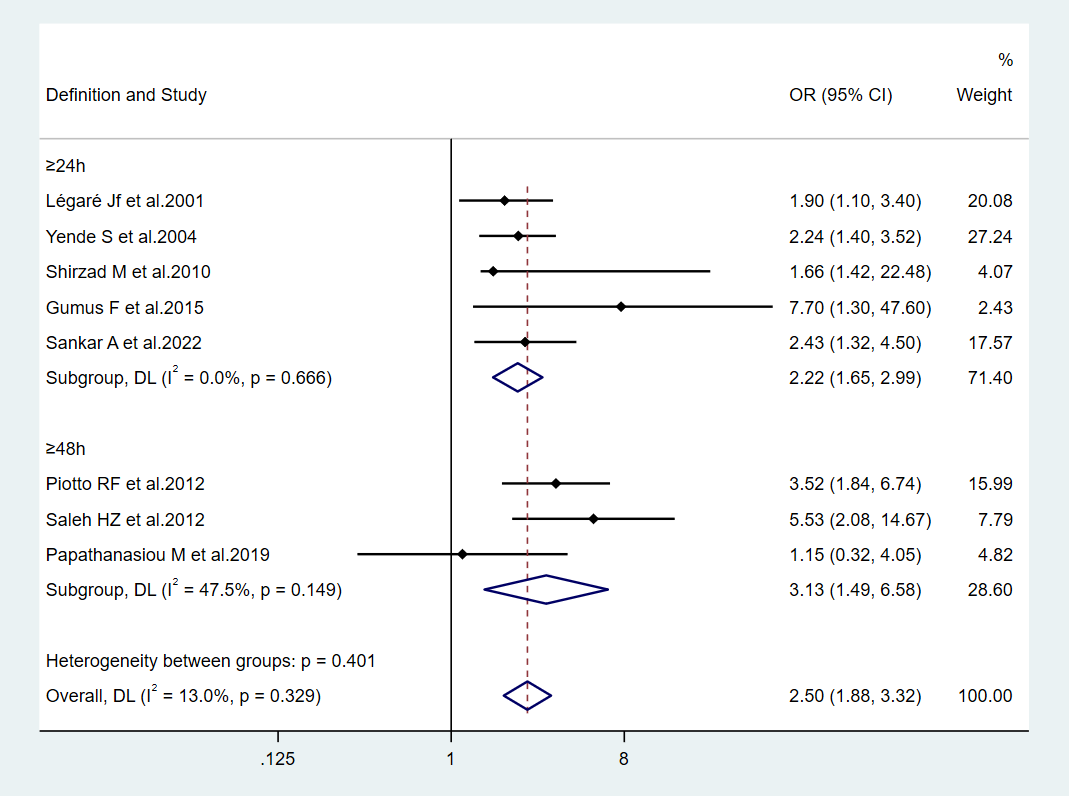


5.7 The subgroup analysis of chronic renal failure.


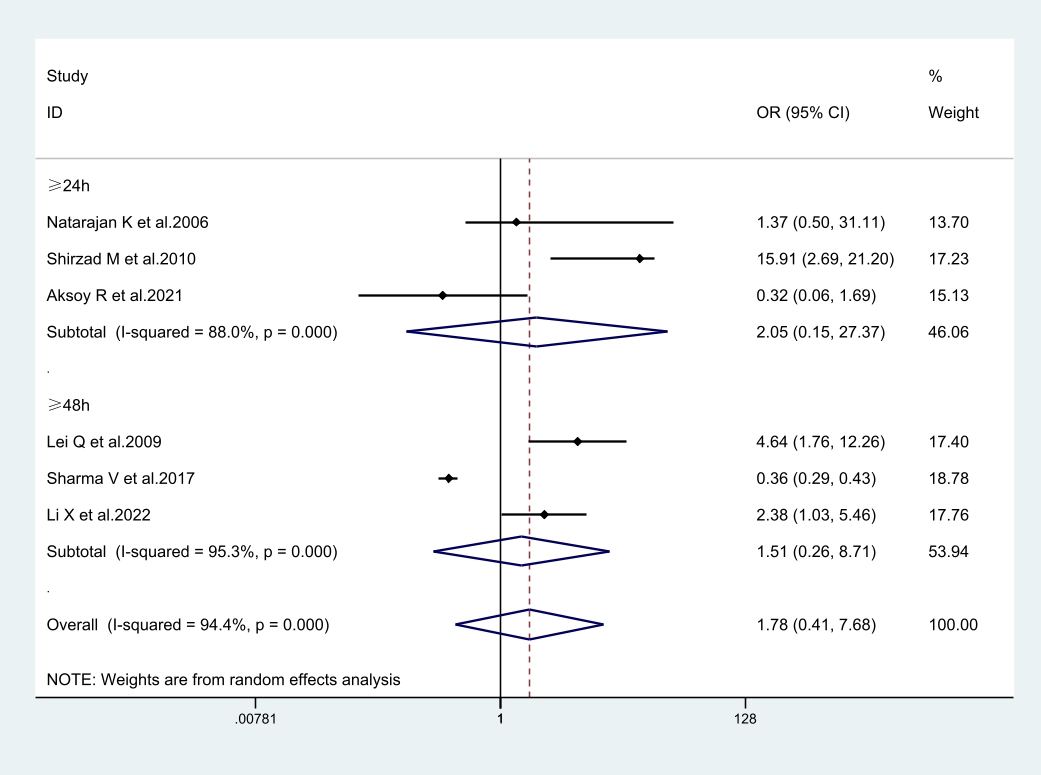


5.8 The subgroup analysis of emergency surgery.


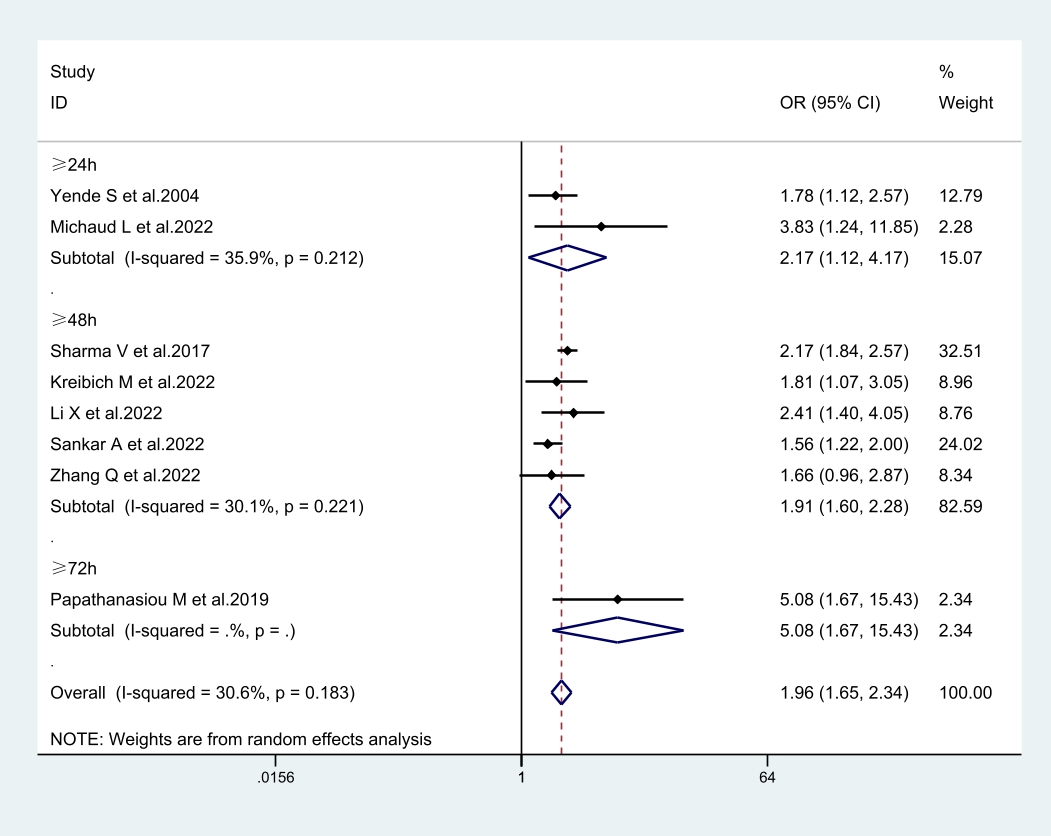


5.9 The subgroup analysis of previous cardiac surgery.


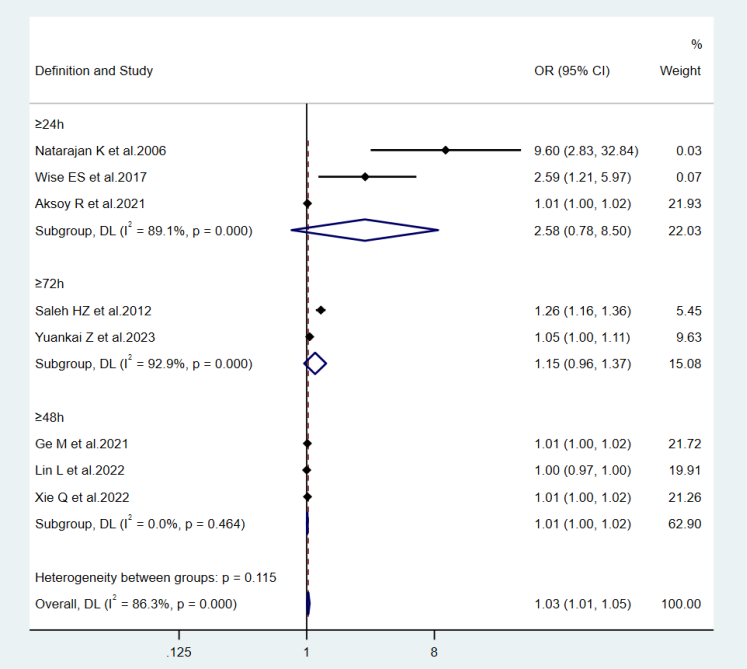


5.10 The subgroup analysis of longer cardiopulmonary bypass time.

**Fig.5 Subgroup analysis of t define of prolonged mechanical ventilation.**


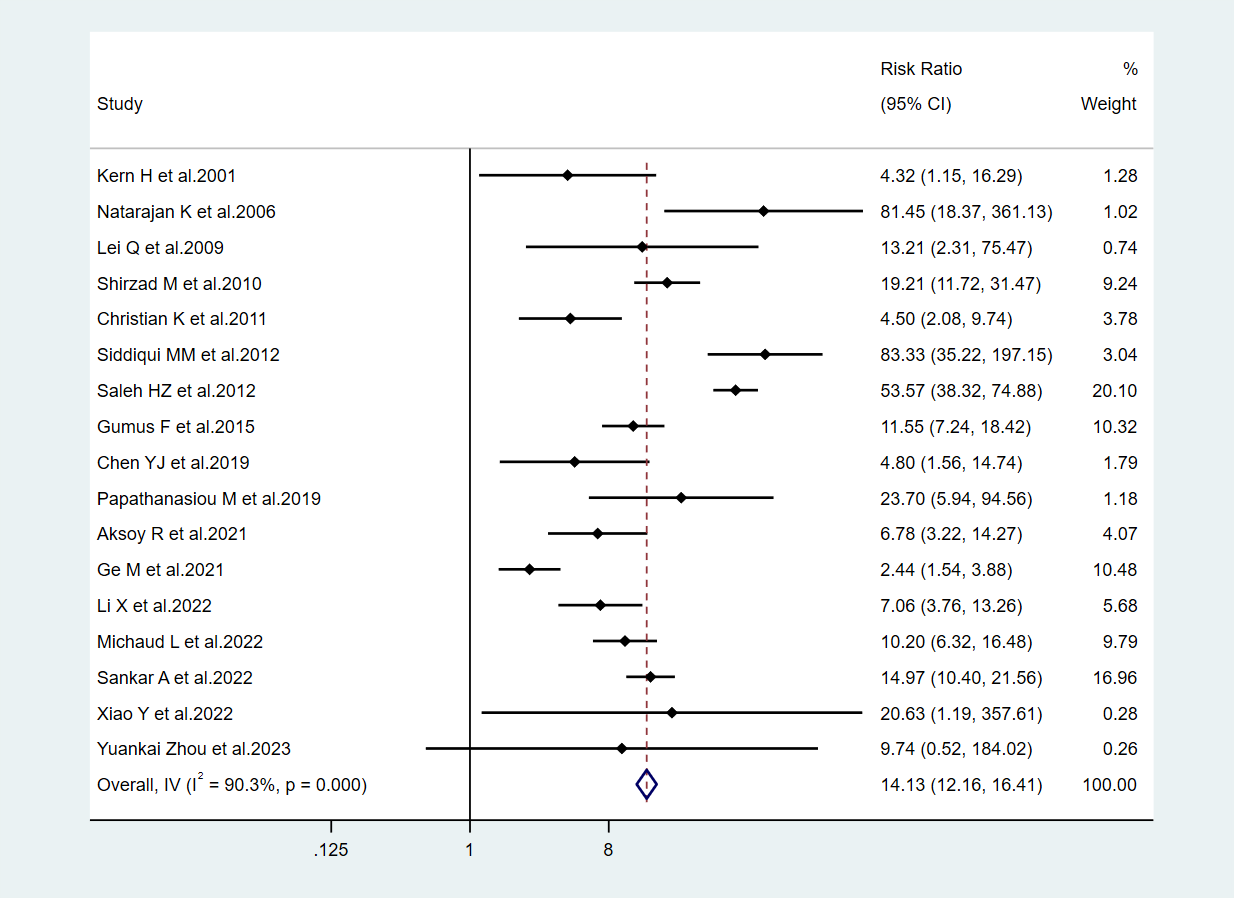


6.1 The forest plot of mortality.


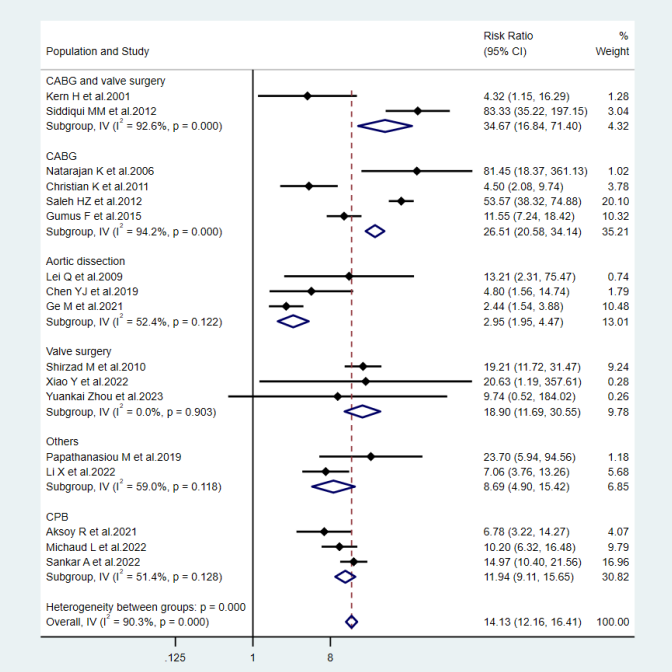


6.2 The subgroup analysis of mortality according to study population.


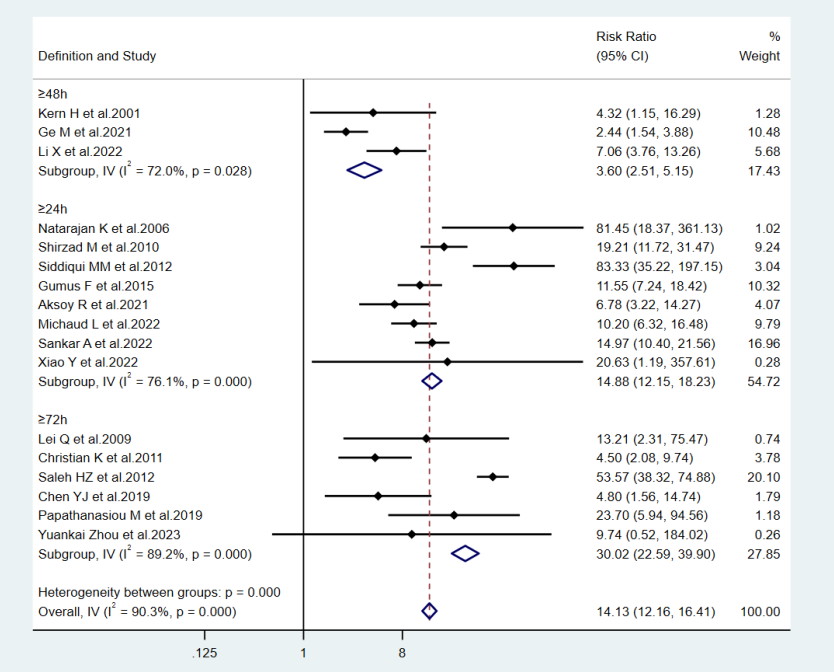


6.3 The subgroup analysis of mortality according to define of prolonged mechanical

ventilation.

**Fig.6 Forest plots for analysis of mortality and its subgroups.**
